# Increased expression of glutathione peroxidase 3 prevents tendinopathy by suppressing oxidative stress

**DOI:** 10.3389/fphar.2023.1137952

**Published:** 2023-03-20

**Authors:** Haruka Furuta, Mari Yamada, Takuya Nagashima, Shuichi Matsuda, Kazuki Nagayasu, Hisashi Shirakawa, Shuji Kaneko

**Affiliations:** ^1^ Department of Molecular Pharmacology, Graduate School of Pharmaceutical Sciences, Kyoto University, Kyoto, Japan; ^2^ Department of Orthopaedic Surgery, Graduate School of Medicine, Kyoto University, Kyoto, Japan

**Keywords:** real-world data, tendinopathy, fluoroquinolone, aging, dexamethasone, GPX3, oxidative stress

## Abstract

Tendinopathy, a degenerative disease, is characterized by pain, loss of tendon strength, or rupture. Previous studies have identified multiple risk factors for tendinopathy, including aging and fluoroquinolone use; however, its therapeutic target remains unclear. We analyzed self-reported adverse events and the US commercial claims data and found that the short-term use of dexamethasone prevented both fluoroquinolone-induced and age-related tendinopathy. Rat tendons treated systemically with fluoroquinolone exhibited mechanical fragility, histological change, and DNA damage; co-treatment with dexamethasone attenuated these effects and increased the expression of the antioxidant enzyme glutathione peroxidase 3 (GPX3), as revealed *via* RNA-sequencing. The primary role of GPX3 was validated in primary cultured rat tenocytes treated with fluoroquinolone or H_2_O_2_, which accelerates senescence, in combination with dexamethasone or viral overexpression of GPX3. These results suggest that dexamethasone prevents tendinopathy by suppressing oxidative stress through the upregulation of GPX3. This steroid-free approach for upregulation or activation of GPX3 can serve as a novel therapeutic strategy for tendinopathy.

## Introduction

The tendon, a fibrous, dense connective tissue, connects muscles with the bone and transmits forces to produce motion. It primarily consists of parallelly arranged collagenous fibers. Tendinopathy is a degenerative disease characterized by pain, loss of strength, or rupture in tendon tissues, accounting for approximately 30% of general practice musculoskeletal consultations (Riley, 2008). Owing to their hypovascularity (Carr and Norris, 1989), which subsequently leads to low nutrition in the tendons and a low turnover rate (Heinemeier et al., 2013), damaged tendons are unlikely to fully recover their original strength (Ohberg et al., 2001). Current treatments for tendinopathy primarily aim to provide symptomatic relief (Kaux et al., 2011), and many documented treatments have failed to accomplish 100% success (Millar et al., 2021).

The pathogenesis of tendinopathy involves multiple intrinsic and extrinsic factors. Aging is one of the major intrinsic causes of tendinopathy. Approximately 50% of individuals in their 70 s suffer from tendinopathy (Kostrominova and Brooks, 2013). Aging lowers metabolism, mechanical properties, and tissue regeneration in tendons (Zhou et al., 2014). Consequently, aged tendons degenerate and sustain injury later in life (Rees et al., 2006), leading to locomotive dysfunction (Minetto et al., 2020). Among extrinsic risk factors, fluoroquinolones have been proven to be associated with tendinopathy (Khaliq and Zhanel, 2003; Tsai and Yang, 2011; Lewis and Cook, 2014). A black box warning label regarding the risk of tendinopathy was added by the Food and Drug Administration (FDA) to all fluoroquinolones in 2008 (Tanne, 2008). However, the precise pathophysiological mechanism of tendinopathy remains unclear.

In this regard, clinical big data analysis has garnered attention as an approach to finding a new therapeutic strategy. The FDA Adverse Event Reporting System (FAERS) is the world’s largest freely available spontaneous reporting system, with more than 10 million cases. The Japanese Adverse Drug Event Report (JADER) and Canada Vigilance Adverse Reaction Online Database (CVARD) are other freely available databases of self-reported adverse events, with approximately 0.7 and 0.9 million reports, respectively. Because these databases contain many cases of polypharmacy, there may be confounding factors that affect the occurrence of drug-induced symptoms. To date, previously unknown drug−drug interactions have been successfully identified by analyzing the information from these databases (Zhao et al., 2013; Nagashima et al., 2016; Nagaoka et al., 2021). However, a major drawback of these databases is the lack of chronological information. To address this challenge and investigate the causal relationship between the use of drugs and tendinopathy, we supplemented the analyzed data from these databases with an analysis of health insurance claims data from the IBM^®^ MarketScan^®^ Research Databases. This database contains daily medical records of diagnosis, treatment, and prescriptions of approximately 44 million patients in the US with Medicare Advantage Plans, employer-sponsored coverage of older individuals, and Medicare supplemental insurance.

We first analyzed the FAERS, JADER, and CVARD databases to explore a negative confounder drug for the occurrence of tendinopathy, focusing on fluoroquinolone-induced tendinopathy. Subsequently, we analyzed the IBM MarketScan data to evaluate the effect of the negative confounder drug on fluoroquinolone-induced and age-related tendinopathy. We then investigated this hypothesis through pharmacological experiments using rats to determine the underlying therapeutic targets and mechanisms. We believe that our findings will help in developing a novel therapeutic strategy for tendinopathy in the future.

## Materials and methods

### Analysis of the spontaneous reporting system database

For the FAERS data, adverse event reports from the first quarter of 2004 to the last quarter of 2019 were obtained from the FDA website (https://www.fda.gov/drugs/drug-approvals-and-databases/fda-adverse-event-reporting-system-faers). Duplicate reports were eliminated as previously reported (Banda et al., 2016), and the remaining 11,438,031 reports were analyzed in this study.

As for the JADER data, adverse event reports from the first quarter of 2004 to the first quarter of 2021 were obtained from the Pharmaceuticals and Medical Devices Agency website (https://www.pmda.go.jp/safety/info-services/drugs/adr-info/suspected-adr/0003.html). A total of 693,295 reports were analyzed in this study.

As for the CVARD data, adverse event reports from January 1965 to April 2021 were obtained from the website of the Government of Canada (https://www.canada.ca/en/health-canada/services/drugs-health-products/medeffect-canada/adverse-reaction-database.html). A total of 880,353 reports were analyzed in this study.

We manually mapped the arbitrary drug names, including trade names and abbreviations, to unified generic names using the Medical Subject Headings descriptor ID. Reports of tendinopathy were defined according to the narrow scope of the standardized MedDRA query (“tendinopathies and ligament disorders”) in MedDRA version 23.0. All analyses were performed as described previously (Zhao et al., 2013). Briefly, adverse event risk was evaluated by calculating the reporting odds ratio (ROR), along with the 95% confidence interval (CI) and *Z* score. Haldane–Anscombe correction was applied to all analyses. In volcano plots, *Z* scores were used instead of *p* values to save space.

### Analysis of the IBM MarketScan research database

The IBM MarketScan Commercial and Medicare Supplemental Databases from January 2017 to December 2019 were purchased from IBM^®^ Watson Health^®^ (Armonk, NY). This dataset contained the medical diagnosis and prescription claims of 43,723,094 employees, retirees, their spouses, and dependents in the United States with primary or Medicare supplemental coverage. The individual diagnoses were assigned according to the International Classification of disease 10 (ICD-10). Definitions of tendinopathy are shown in Supplementary Table S1. To identify patients who were prescribed fluoroquinolones, we defined the fluoroquinolone cohort as the patients who received drugs belonging to the ATC code J01MA (Supplementary Table S1). The patients who received only topical fluoroquinolones were excluded. Similarly, the dexamethasone cohort comprised patients who received drugs belonging to ATC code H02AB02 (Supplementary Table S1), and the patients who received dexamethasone for only topical usage were excluded.

After examining the time distribution of the first event after enrollment in IBM, we defined the run-in period to detect the onset and causality of tendinopathy by eliminating the patients who had already received a diagnosis of tendinopathy or a prescription of fluoroquinolone or dexamethasone before the enrollment for insurance.

A 1:1 propensity score matching was used to balance known confounding variables in tendinopathy; covariates such as demographics, comorbidities, medication use, and history of hospitalization were controlled. Propensity score-matched pairs were created by matching two groups using the nearest-neighbor method with a 0.2 caliper width in the fluoroquinolone cohort and the older adult cohort. A Chi-square test was used to compare comorbidities, and a Wilcoxon rank sum test or Welch’s t-test was used to compare age, as shown in the results section (propensity score matching).

Once the differences between the cohorts were well-balanced, the daily and cumulative doses and administration periods of fluoroquinolones and dexamethasone were quantified and compared. Cumulative incidences of tendinopathy were compared between the cohorts using conventional survival analysis (Yokoyama et al., 2018), and the survival curves were represented by Kaplan–Meier plots. Statistical significance was evaluated using the log-rank test and Cox proportional regression analysis to calculate the hazard ratios. The number at risk indicated the number of patients who may have had an onset of tendinopathy each day.

Analyses of the IBM MarketScan data were performed using R v4.1.0 and R studio 2022.02.3 software (R Foundation for Statistical Computing, Vienna, Austria), with R package survival for the time-series analysis and SAS OnDemand for Academics (SAS Institute, Cary, NC) for propensity score matching.

### Animals

All animal experiments were conducted in accordance with the ethical guidelines and approved by the Kyoto University Animal Research Committee (Approval No. 19–38). All experiments were designed to minimize the use of animals and the number of experiments performed. Male Wistar/ST rats (10-week-old for *in vivo* experiments and 6- to 8-week-old for cell isolation) were purchased from Japan SLC (Shizuoka, Japan). All animals were housed at a constant ambient temperature (22°C ± 2°C) on a 12-h light/dark cycle, with free access to food and water. Pefloxacin (LKT Laboratories, St. Paul, MN) and dexamethasone (Nacalai Tesque, Kyoto, Japan) were suspended in 0.5% carboxymethyl cellulose. Pefloxacin was administered to rats (900 mg/kg/day, 5 days a week for 4 weeks and drug withdrawal for 1 week) to develop a tendinopathy model. In a previous report, 2-week administration of pefloxacin (900 mg/kg/day) to rats caused the emergence of tenocytes with round nuclei in Achilles tendons (Kato et al., 1995), which was also observed in Achilles tendons of patients with tendinopathy (de Mos et al., 2009); however, biomechanical testing was not performed in that study. We slightly modified that treatment schedule to induce mechanical fragility of the Achilles tendon. Dexamethasone was concomitantly administered orally (50 µg/kg/day, 3 days a week for 5 weeks) to evaluate its effect on fluoroquinolone-induced tendinopathy. Dexamethasone was administered once every 2–3 days as its biological half-life is 36–54 h.

### Biomechanical examination

Biomechanical examination of the rats was performed using a tensile testing machine (MODEL-1310VRW, Aikoh Engineering, Osaka, Japan). The Achilles tendon was fixed using metal clamps and connected to the pulling device. The test velocity was maintained at 50 mm/min. The point of rupture of the Achilles tendon was monitored and recorded on a computer screen. At the end of the test, strength-elongation graphics were obtained and documented. The diameter of the Achilles tendon was measured using a vernier caliper, and maximum stress was calculated from the test results.

### Hematoxylin and eosin staining

After rats were sacrificed, Achilles tendons were fixed in 4% paraformaldehyde for 48 h. After fixation, tissues were dehydrated and embedded in paraffin. For descriptive histology, 5 μm-thick sections were stained with hematoxylin and eosin. Nuclei were stained purple, and those with a minor axis length of more than 4 µm were counted as cells with round nuclei.

### RNA sequencing

Total RNA was isolated from the Achilles tendon using the ISOGEN reagent (Nippon Gene, Tokyo, Japan). For RNA-sequencing (RNA-seq) analysis, poly(A)^+^ RNA was selected from the total RNA and sequenced using DNBseq (BGI, Shenzhen, China). The clean reads were mapped to the rat reference genome GCF_000001895.5_Rnor_6.0, and the reference gene sequences were mapped using HISAT version 2.0.4 (Kim et al., 2015) and Bowtie2 version 2.2.5 (Langmead and Salzberg, 2012). Gene expression was calculated using RSEM version 1.2.8 (Li and Dewey, 2011) and subsequently log-transformed (for example, the expression level of 8 (=2^3^) becomes 3, the expression level of 128 (= 2^7^) becomes 7, and so on). We applied the Gene ontology analysis to uncover the biological processes related to tendinopathy. Statistical significance was set at *p* < 0.05. DAVID v6.8, a web-based bioinformatics resource intended for functional genomics analysis, was used to analyze genes whose expression levels, with transcripts per million (TPM) ≥ 100 in the vehicle group, significantly increased in the pefloxacin-treated group compared with those in the vehicle group and those whose expression levels decreased in the pefloxacin + dexamethasone group compared with those in the pefloxacin group. *Z* scores across treatment groups were visualized as a heatmap using Prism v 9.4.0. The datasets generated from RNA-seq are available under the Gene Expression Omnibus (GEO) accession code GEO: GSE212344.

### Analysis of gene expression omnibus

The dataset for gene expression in human tendinopathy and normal tendon tissue was downloaded from the GEO accession GSE26051 (Jelinsky et al., 2011). Expression data were available for 23 samples from patients with tendinopathy and 23 normal samples.

### Cell isolation and culture

Male Wistar ST rats (6- to 8-week-old) were sacrificed by CO_2_ asphyxiation, and their Achilles tendons were removed and cut into approximately 1 mm^3^ pieces. These were cultured with Dulbecco’s modified Eagle’s medium (D5796, Sigma-Aldrich, Saint-Louis, MO) supplemented with 10% heat-inactivated fetal bovine serum (Sigma-Aldrich), 100 U/mL penicillin, and 100 μg/mL streptomycin (Nacalai Tesque). The cultures were maintained at 37°C in a 5% CO_2_ humidified atmosphere. After 7–21 days, tenocytes were observed in the cultures and grown till they reached 90% confluence. They were sub-cultured, and tenocytes between passages 3–10 were used for further experiments. After seeding the cells for drug treatments, they were kept in a 2% O_2_ atmosphere to mimic the tendon environment. The medium was changed to a serum-free medium (D6046, Sigma-Aldrich) 48 h after seeding, and sodium selenite (100 nM, S9133, Sigma-Aldrich) was added at the same time for maintaining the expression of GPX3.

### RNA isolation and quantitative reverse transcription-polymerase chain reaction

Total RNA was isolated using the ISOGEN reagent (Nippon Gene) and reverse transcribed to cDNA using the ReverTra Ace qPCR RT Kit (Toyobo, Osaka, Japan). Quantitative reverse transcription polymerase chain reaction (qRT-PCR) was performed using the StepOne Real-Time PCR System (Thermo Fisher Scientific, Waltham, MA) and THUNDERBIRD SYBR qPCR Mix (Toyobo). PCR amplification consisted of heat activation for 10 min at 95°C, followed by 40 cycles at 95°C for 15 s and 60°C for 1 min. The primer sets used for each gene are listed in Supplementary Table S2.

### Immunofluorescence

For *in vivo* study, the Achilles tendons were fixed with 4% paraformaldehyde (Nacalai Tesque) in phosphate buffer. Subsequently, this fixed tissue was embedded in the Tissue-Tek OCT compound (Sakura Finetek, Tokyo, Japan), frozen, and stored at −80°C for cryosectioning. The tendon was cryosectioned into 14 µm-thick sections with a cryostat (Leica CM1950; Leica Biosystems, Vista, CA) and stored again at −80°C. The sections were immersed in phosphate-buffered saline containing 0.1% Triton X-100 (Nacalai Tesque) for permeabilization and incubated with rabbit polyclonal anti-phospho-histone H2A.X (γH2AX) antibody (1:300; #2577, Cell Signaling Technology, Danvers, MA) overnight at 4°C, followed by incubation with Alexa Fluor 594-labelled donkey anti-rabbit IgG (1:300; A21207, Thermo Fisher Scientific) for 2 h at room temperature in the dark. Nuclei were stained with DAPI Fluoromount-G (SouthernBiotech, Birmingham, AL).

For *in vitro* study, the cells were fixed with 4% paraformaldehyde in phosphate buffer. Next, they were immersed in phosphate-buffered saline containing 0.1% Triton X-100 for permeabilization and incubated with rabbit polyclonal anti-γH2AX antibody (1:300; #2577, Cell Signaling Technology) overnight at 4°C, followed by incubation with Alexa Fluor 594-labelled donkey anti-rabbit IgG (1:300; A21207, Thermo Fisher Scientific) for 2 h at room temperature in the dark. Nuclei were stained with DAPI Fluoromount-G (SouthernBiotech).

Images were captured using a confocal fluorescence microscope (FluoView FV10i; Olympus, Tokyo, Japan). The number of γH2AX^+^ cells was counted in 0.045 and 0.10 mm^2^ fields for *in vivo* and *in vitro* studies, respectively.

### Measurement of reactive oxygen species

Tenocytes were stained for reactive oxygen species (ROS) using the CellROX Deep Red Reagent (C10422, Thermo Fisher Scientific) for measuring ROS according to the manufacturer’s protocol. Nuclei were stained with DAPI Fluoromount-G. Images were captured using a confocal fluorescence microscope (FluoView FV10i; Olympus). The mean fluorescence intensity of the ROS signal of cells in a 0.18 mm^2^ field was calculated using ImageJ (version 1.51m9, National Institutes of Health, Bethesda, MD).

### Senescence-associated β-galactosidase (SA-β-gal) staining

SA-β-gal staining was performed according to the manufacturer’s instructions (#9860, Cell Signaling Technology). After drug treatment, tenocytes were incubated with the staining solution provided in the kit for 16 h at 37°C. The percentage of SA-β-gal^+^ cells in a 0.14 mm^2^ field was calculated.

### Vector construction

For constructing pCSII-CAG-Venus-WPRE, a CAG fragment was amplified *via* PCR and ligated into pCSII-EF-Venus-WPRE [gifted by Dr Miyoshi and Dr Miyawaki (RIKEN, Japan) (Nagai et al., 2002)]. For constructing pCSII-CAG-GPX3-WPRE, pCSII-CAG-Venus-WPRE was ligated with a GPX3 fragment amplified *via* PCR from the cDNA of the Achilles tendon of a rat. PCR was performed with Q5 DNA polymerase (New England Biolabs, Ipswich, MA). All ligation reactions were performed using Ligation high Ver. 2 (Toyobo). The constructs were verified using Sanger sequencing (Eurofins Genomics, Tokyo, Japan). The primers used for amplification are shown in Supplementary Table S2. The plasmids generated in this study will be made available upon request.

### Lentiviral production and infection

A self-inactivating lentiviral vector (Miyoshi et al., 1998) was prepared and purified. Lenti-X 293T cells (Clontech, Mountain View, CA) were grown to 60%–70% confluency, and the packaging vectors and shuttle constructs were transfected with polyethyleneimine (Polysciences, Warrington, PA). After 16–18 h of incubation, the supernatant was aspirated, and a fresh medium was added to the culture. After 30 h of incubation, the supernatant was collected and filtered through a 0.45 µm-pore size PVDF membrane (Millex-HV, Merck Millipore, Billerica, MA), and lentiviral transfection was conducted on tenocytes. These transfected tenocytes were used in bulk for experiments.

### Statistical analysis

Statistical analyses of *in vivo* and *in vitro* results were performed using Prism v9.4.0 (GraphPad). Sidak’s multiple comparison test was used for *post hoc* analysis. Statistical significance was set at *p* < 0.05. Data were presented as mean ± SEM.

## Results

### Drug-induced tendinopathy in spontaneous reporting systems

**FIGURE 1:**
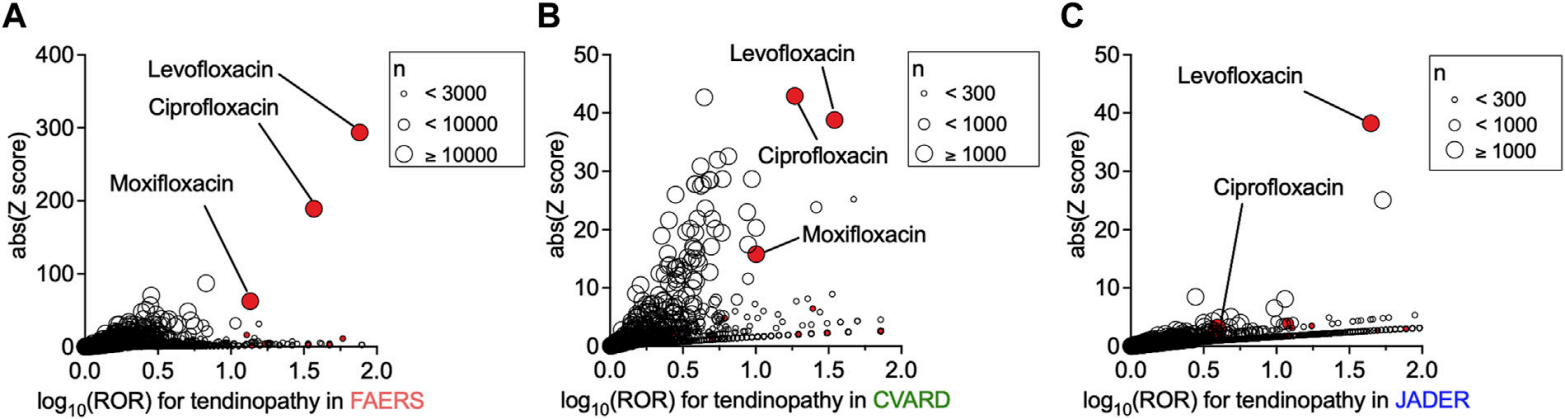
Increased incidence of tendinopathy with the prescription of drugs in patients registered in the FDA Adverse Event Reporting System (FAERS), Canada Vigilance Adverse Reaction Online Database (CVARD), and Japanese Adverse Drug Event Report (JADER). The precise equations used in the analyses, all drugs, reporting odds ratio (ROR) values, and *Z* scores are presented in Supplementary Data S1. Volcano plots of ROR on a log scale and statistical significance (absolute *Z* score) are shown. Each circle indicates an individual drug, and the size of the circle reflects the number of patients taking the drug. A strong and significant increase in the ROR of tendinopathy was observed in patients registered in FAERS **(A)**, CVARD **(B)**, and JADER **(C)** taking fluoroquinolones (red plots), such as levofloxacin, ciprofloxacin, and moxifloxacin.

First, we investigated the association between the use of a particular drug and the incidence of tendinopathy in FAERS, CVARD, and JADER using a disproportionality analysis by calculating each ROR and its *Z* score. Owing to known reporting biases and the absence of incidence denominators accompanying self-reports, these values may not reflect the actual incidence rate. Nevertheless, the use of many fluoroquinolones showed strong associations with the occurrence of tendinopathy, with high RORs and *Z* scores in all three datasets (Figures 1A–C and Supplementary Data S1). These findings confirmed that the positive correlation between fluoroquinolones and tendinopathy was reflected in the databases.

### Confounding effects of dexamethasone on the ROR of fluoroquinolone-induced tendinopathy

**FIGURE 2:**
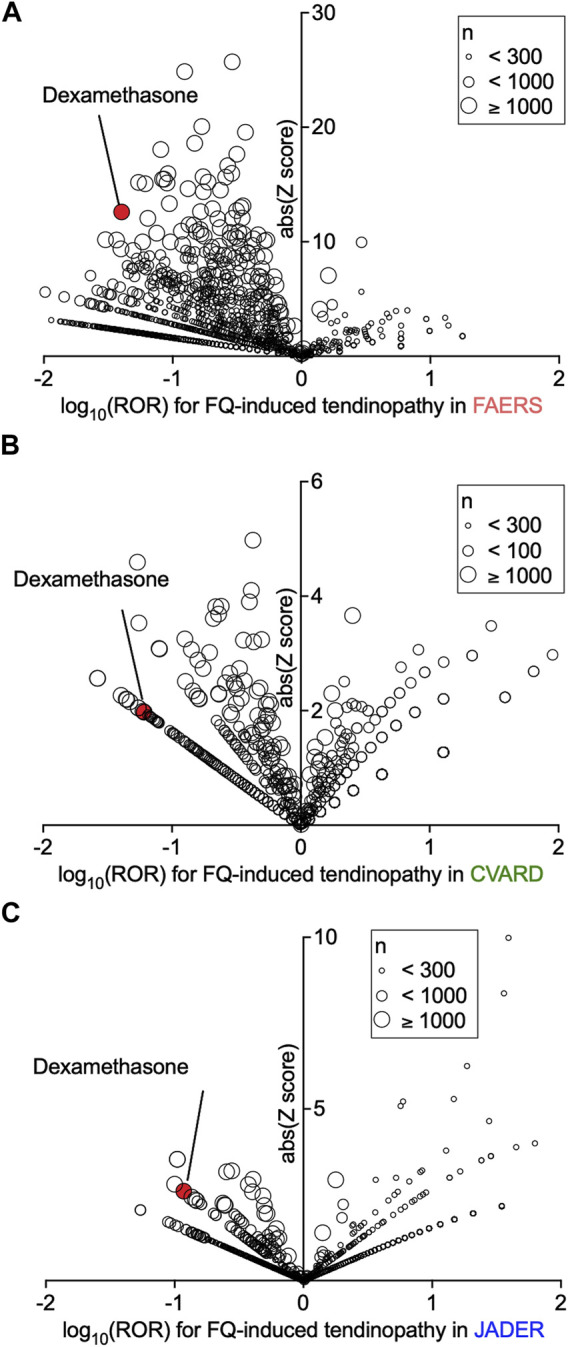
Confounding effects of concomitant drugs on fluoroquinolone-induced tendinopathy in patients registered in the FDA Adverse Event Reporting System (FAERS), Canada Vigilance Adverse Reaction Online Database (CVARD), and Japanese Adverse Drug Event Report (JADER). The confounding effects of all drug combinations on the reporting odds ratio (ROR) for tendinopathy were calculated. Volcano plots visualizing the ROR (on a log scale) and its statistical significance (absolute *Z* score) from FAERS **(A)**, CVARD **(B)**, and JADER **(C)** are shown. The overall values are presented in Supplementary Data S2.

Evaluation of confounding effects of drug combinations on the ROR of tendinopathy in a population of fluoroquinolone users in FAERS, JADER, and CVARD revealed that many concomitantly used drugs affected the ROR of fluoroquinolone-induced tendinopathy (Figures 2A–C and Supplementary Data S2). We assumed that the drugs exhibiting a strong negative correlation with tendinopathy were more likely to be enriched from among the drugs that significantly decreased the ROR values in all three datasets. Notably, six drugs were enriched, and among them, dexamethasone showed a distinctly small ROR value in all datasets (Supplementary Table S3). Many other systemic steroids decreased or unaltered the occurrence of tendinopathy; their ROR values were not as small as that of dexamethasone, in parallel with the strength of the glucocorticoid effect (Supplementary Table S4).

However, in 2008, FDA sent out a warning that steroids increased the risk of fluoroquinolone-induced tendinopathy (Tanne, 2008). Our finding was opposite to that of the FDA’s warning. Hence, we decided to further investigate this conflicting finding regarding the effect of steroids, focusing on dexamethasone.

### Effect of dexamethasone on the incidence rate of fluoroquinolone-induced and age-related tendinopathy in the IBM MarketScan data

Next, we analyzed IBM MarketScan data to conduct a time-series analysis, first examining the effect of dexamethasone on fluoroquinolone-induced tendinopathy. The time distribution assessment of the first event after enrolling patients in the IBM MarketScan database indicated that the number of patients who were initially diagnosed with tendinopathy or prescribed fluoroquinolone or dexamethasone was much higher during the first 5 months than in the subsequent months and stabilized after 6 months (Supplementary Figure S1). These results suggested that a considerable number of patients were diagnosed with tendinopathy or prescribed fluoroquinolone or dexamethasone before or just after enrollment. Therefore, to evaluate the timeline of fluoroquinolone-induced tendinopathy, data of patients diagnosed with tendinopathy or receiving a prescription for fluoroquinolone or dexamethasone during the first 0- to 5-month run-in period were excluded.

**FIGURE 3:**
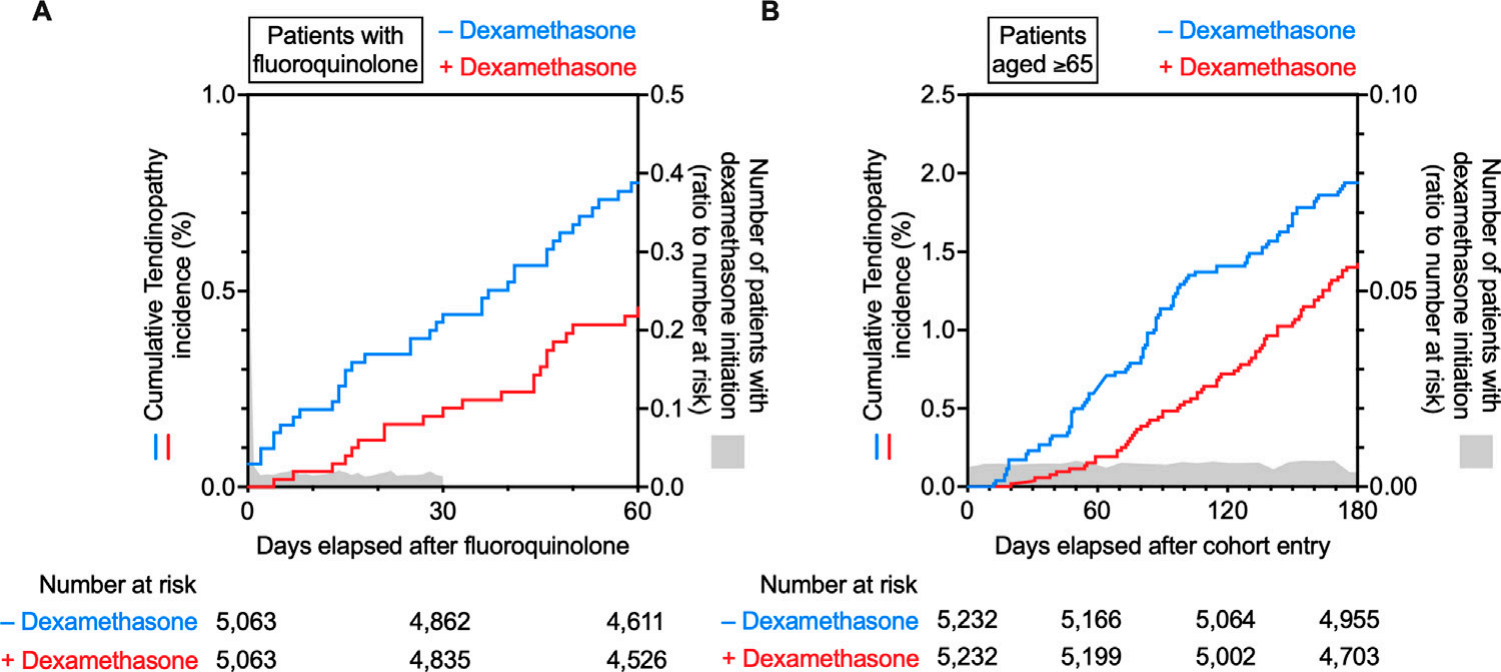
Time trends in the incidence of tendinopathy in the fluoroquinolone-prescribed or older adult cohort in the IBM MarketScan data. Kaplan–Meier curves for the cumulative incidence ratio of tendinopathy in patients taking fluoroquinolone **(A)** or patients aged ≥65 years **(B)** are shown individually as two populations: one without (blue) and one with (red) co-prescribed dexamethasone. The number of patients with dexamethasone initiation is shown in gray as a ratio to the number at risk.

To ensure an overlap between the dosing period of dexamethasone and the risk period of fluoroquinolone-induced tendinopathy, we first assessed the data of dexamethasone prescribed before fluoroquinolone. The median dosing period per prescription was 5 days (interquartile range: 3–12 days). Because the biological half-life of dexamethasone is 36–54 h, we considered overlapping prescriptions, wherein dexamethasone was prescribed within 7 days before the prescription of fluoroquinolone. Furthermore, according to reports, 85% of patients experienced tendinopathy within 1 month after fluoroquinolone treatment, and 41%–50% of patients presented with symptoms after discontinuation of fluoroquinolone (Lewis and Cook, 2014). Therefore, we set the risk period of tendinopathy to 30 days after the prescription of fluoroquinolone. Dexamethasone prescribed in that period was also regarded as an overlapping prescription. Altogether, dexamethasone prescribed from 7 days before to 30 days after the prescription of fluoroquinolone was defined as concomitant use. According to this definition, we divided the fluoroquinolone cohort into two groups, those treated with concomitant dexamethasone (*n* = 5,063) and those that were not (*n* = 1,562,000). Subsequently, 1:1 PSM was conducted to minimize the effects of confounding variables that could influence tendinopathy occurrence (Seeger et al., 2006; Abate et al., 2009; Bolon, 2017; Millar et al., 2021) and optimize the type of fluoroquinolone. After PSM, the two cohorts achieved an adequate balance of the measured covariates and type of fluoroquinolone. Between these matched cohorts, there was no remarkable difference in the dosage of fluoroquinolone (Supplementary Table S5). The median administration period of dexamethasone was 12 days. The main diagnosis of patients when dexamethasone was prescribed was cancer, suggesting that dexamethasone was used as an antiemetic. Patients were followed up for 60 days after prescribing fluoroquinolone, i.e., double the length of the risk period. Kaplan-Meier analysis and Cox proportional hazards modelling indicated that dexamethasone mitigated the risk of fluoroquinolone-induced tendinopathy with a hazard ratio of 0.58 (Figure 3A, 95% CI: 0.34–0.98, *p* = 0.04 in the log-rank test). In five patients who did not receive dexamethasone treatment, a severe phenotype accompanied by tendon tear or rupture was observed, and the total incidence proportion declined from 0.75% to 0.43% with dexamethasone (Supplementary Table S5).

Next, we tested the efficacy of dexamethasone against age-related tendinopathy. Similarly to the analysis of fluoroquinolone-induced tendinopathy, patients diagnosed with tendinopathy or receiving dexamethasone during the first 0- to 5-month run-in period were excluded from this analysis. Individuals aged 65 years or older, registered in the IBM MarketScan data, were divided into two groups: those who received dexamethasone (*n* = 5,232) and those who did not (*n* = 1,538,993). Then, PSM was performed to eliminate known confounding factors for tendinopathy (Supplementary Table S6). Patients were followed up for 180 days from the end of the wash-out period. A comparison of the two cohorts using Kaplan-Meier analysis and Cox proportional hazards modelling revealed that dexamethasone mitigated the incidence of age-related tendinopathy with a hazard ratio of 0.72 (Figure 3B, 95% CI: 0.53–0.98, *p* = 0.03 in the log-rank test). Dexamethasone was presumably prescribed as an antiemetic based on the diagnoses given to the patients with its prescription. The median administration period of dexamethasone was only 10 days (Supplementary Table S6). Dexamethasone reduced the incidence proportion in more than half of the types of tendinopathy categorized in ICD-10, and the total incidence proportion of tendinopathy decreased from 2.14% to 1.45% (Supplementary Table S6).

These results from the analyses of real-world patient data suggested that dexamethasone prevented the onset of tendinopathy induced by fluoroquinolone and aging.

### Effects of dexamethasone on fluoroquinolone-induced tendinopathy in rats

**FIGURE 4:**
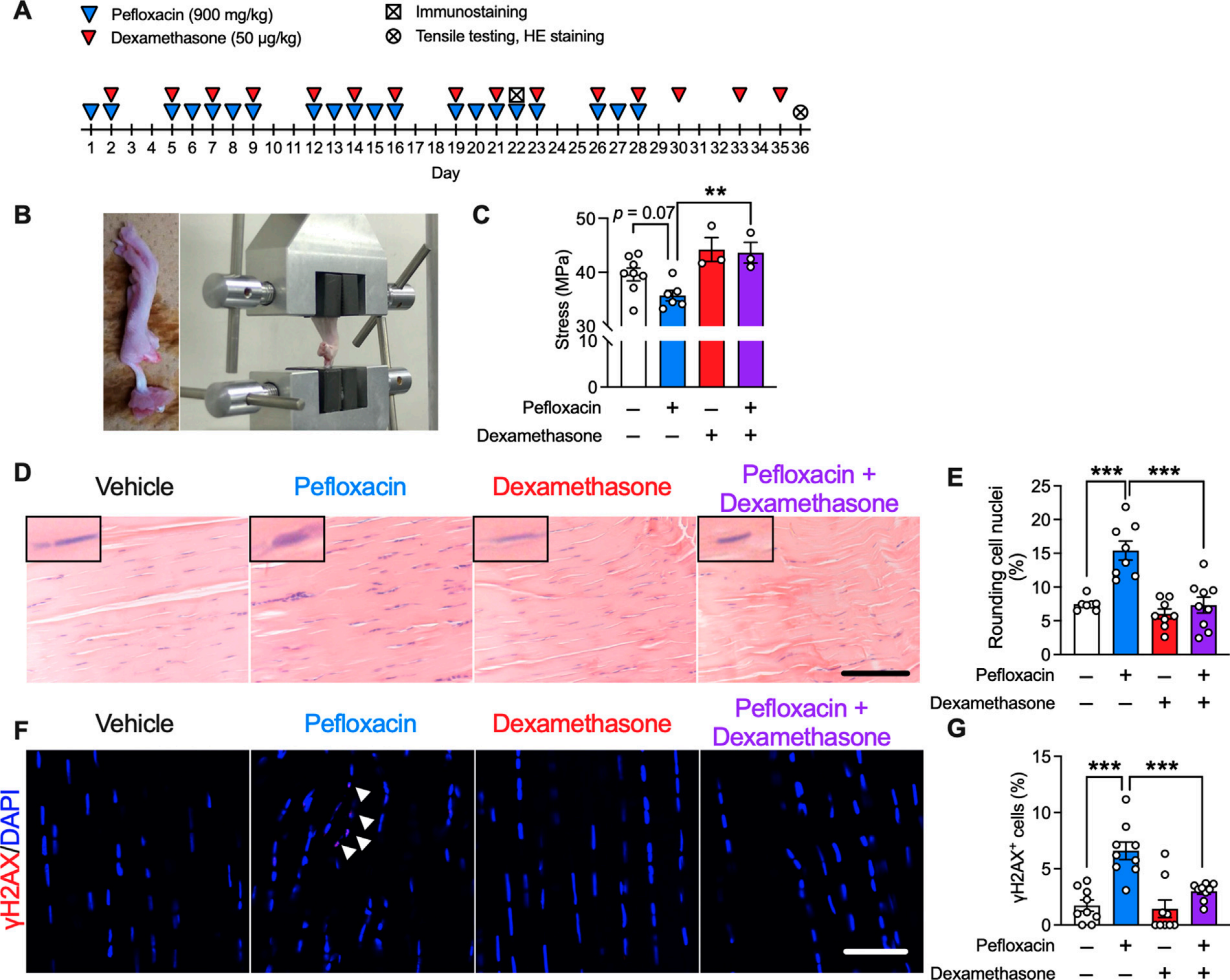
Effects of dexamethasone on a pefloxacin-induced tendinopathy rat model. **(A)** Rats were treated with pefloxacin (900 mg/kg/day, 5 days a week for 4 weeks), dexamethasone (50 µg/kg/day, 3 days a week for 5 weeks), or both, and Achilles tendons were collected 1 day after completing the 3-week administration for immunostaining and 1 day after completing the 5-week administration for tensile testing and hematoxylin and eosin staining (HE staining). **(B)** Images of biomechanical testing. **(C)** Biomechanical properties were examined immediately after the collection of Achilles tendons from drug-administered rats (*n* = 3–8 per group). Maximum stress was calculated from test results. **(D,E)** Representative images of HE staining (*n* = 7–9 per group) with representative nuclei in the upper left of each picture; summarized data for cells with round nuclei. Scale bar: 0.1 mm. **(F,G)** Immunostaining of γH2AX was performed, and the samples were imaged using confocal microscopy (*n* = 9 per group). The number of γH2AX^+^ cells (shown with the arrowhead) is presented as a percentage of the number of total cells. Scale bar: 50 µm. Data are shown as mean ± SEM. Statistical significance was tested using a two-way ANOVA with *post hoc* multiple comparisons; **p < 0.01, ***p < 0.001.

To test the hypothesis derived from the human data, we developed a rat tendinopathy model by chronic oral administration of pefloxacin, and dexamethasone was co-administered (Figure 4A). Tensile testing was performed to evaluate the biomechanical properties of the Achilles tendon. Compared to that of the vehicle group, chronic treatment with pefloxacin tended to decrease the maximum stress of the Achilles tendon, which was reversed by the co-administration of dexamethasone (Figures 4B, C). Histologically, chronic pefloxacin treatment increased the ratio of cells with round nuclei, a phenomenon seen in tendinopathy (Kato et al., 1995; de Mos et al., 2009), which was suppressed by co-treatment of dexamethasone, and no infiltration of inflammatory cells was observed (Figures 4D, E). Meanwhile, dexamethasone alone did not change the maximum stress of the tendon or the ratio of cells with round nuclei. Previous studies reported DNA damage in response to fluoroquinolone use (Li et al., 2010), overuse (Thirupathi et al., 2018), and aging (Yousefzadeh et al., 2021), all of which are risk factors for tendinopathy. Therefore, we investigated DNA damage in the Achilles tendon by immunostaining for γH2AX, a sensitive marker of DNA damage. DNA damage persists for 1 day but is most likely to be repaired within 3 days (Tryfidou et al., 2020); hence, DNA damage was evaluated not a week after the end of 4-week pefloxacin administration but a day after 3 weeks of drug administration. Thus, many γH2AX^+^ cells were observed in the Achilles tendon of rats administered pefloxacin, which was mitigated by dexamethasone co-treatment. Notably, dexamethasone alone did not affect the number of γH2AX^+^ cells (Figures 4F, G).

### Molecular mechanisms underlying the effect of dexamethasone on pefloxacin-induced tendinopathy

**FIGURE 5:**
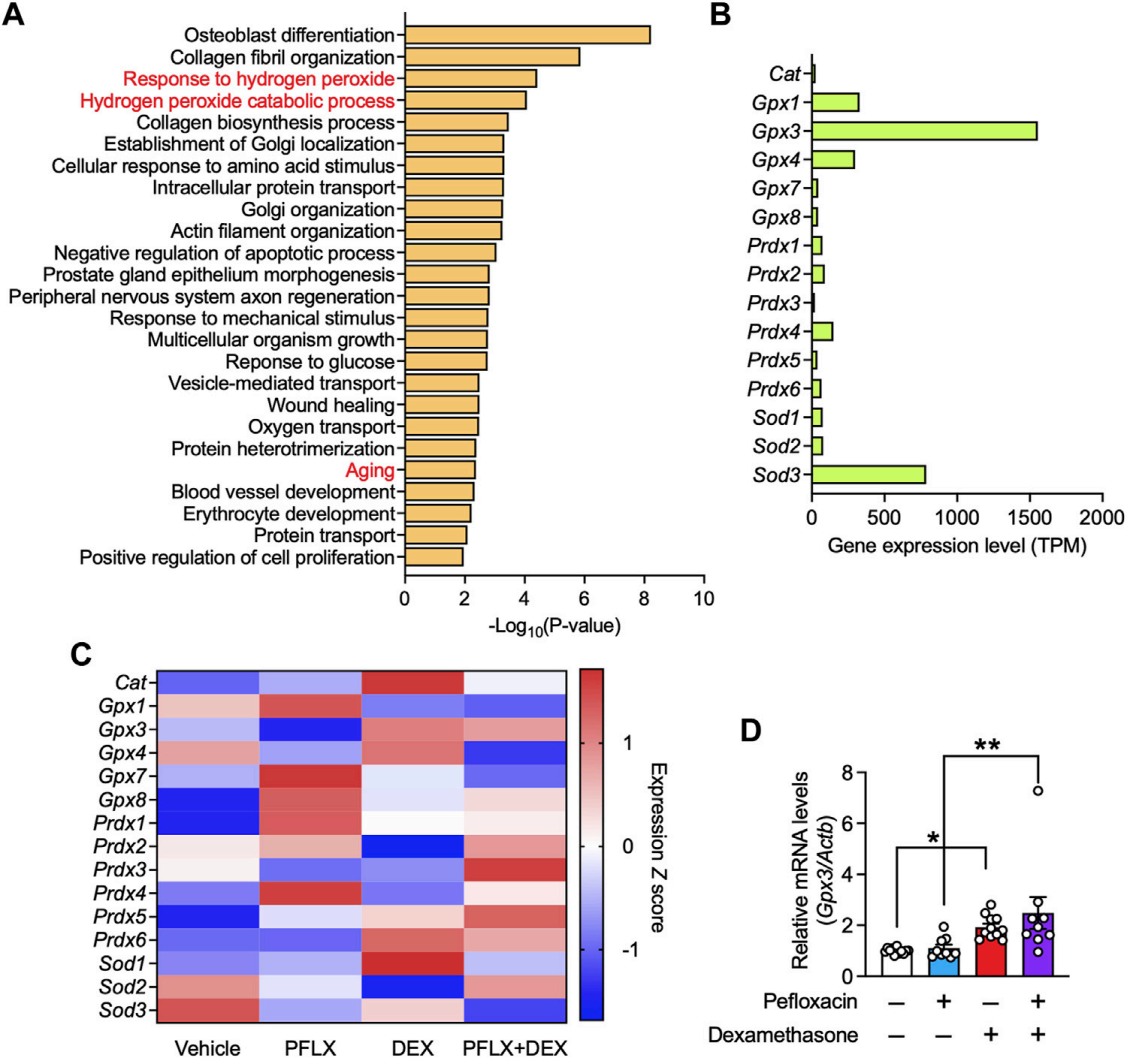
Gene expression in the tendon tissues of rats treated with pefloxacin, dexamethasone, or both. **(A)** Gene ontology (GO) analysis of genes whose expression, with transcripts per million (TPM) ≥ 100 in the vehicle group, was increased in the pefloxacin group compared with that in the vehicle group and reduced in the group administered both pefloxacin and dexamethasone compared with that in the pefloxacin group. The top 25 significantly enriched GO terms are shown. **(B)** The relative gene expression levels, shown as TPM, of antioxidant enzymes in the Achilles tendon from a vehicle-administered rat. **(C)** Heatmap of gene expression of antioxidant enzymes in Achilles tendon from rats administered vehicle, pefloxacin (PFLX), dexamethasone (DEX), or both (PFLX + DEX). Red and blue colors represent large and small standardized values in *Z* scores, respectively. **(D)** The gene expression of *Gpx3* was examined using quantitative RT-PCR (*n* = 9–12 per group) and represented as the relative ratio to the control group. Data are shown as the mean ± SEM. Statistical significance was tested using a two-way ANOVA with *post hoc* multiple comparisons; *p < 0.05, **p < 0.01.

To investigate the underlying molecular mechanism, we conducted RNA-seq of samples recovered from the Achilles tendons of drug-administered rats. Gene ontology analysis using the DAVID tool, focusing on the genes upregulated by pefloxacin and whose upregulation was suppressed by dexamethasone co-administration, revealed that several pathways related to oxidative stress, such as “response to hydrogen peroxide” and “hydrogen peroxide catabolic process,” were enriched (Figure 5A). No pathways associated with inflammation were enriched in this analysis. Notably, “aging” was also enriched. This result suggested that oxidative stress was increased by fluoroquinolone and suppressed by co-treatment with dexamethasone. Additionally, it indicated that the expression levels of some age-related molecules were increased by pefloxacin treatment, and this effect was mitigated by dexamethasone.

Next, we focused on antioxidant enzymes because they primarily regulate the level of ROS, a major cause of oxidative stress. The gene expression of all antioxidant enzymes in the Achilles tendon of vehicle-treated rats was investigated, and some glutathione peroxidases (*Gpx1, Gpx3,* and *Gpx4*) and superoxide dismutase 3 (*Sod3*) were found to have high expression levels (Figure 5B). Then, the changes in their expression levels after drug administration were analyzed. Pefloxacin treatment reduced the gene expression of several antioxidant enzymes, including *Gpx3*, *Gpx4, Sod2*, *Sod3*, and peroxiredoxin 3 (*Prdx3*); co-treatment with dexamethasone mitigated the reduced expression of some genes Figure 5C. GPX3 was identified as a potential target, with the highest expression level among the antioxidant enzymes examined in the rat Achilles tendon. The expression level of *Gpx3* was validated in the specimen using qRT-PCR. Although the downregulation of *Gpx3* by pefloxacin was not observed, Achilles tendons from rats treated with only dexamethasone or with both pefloxacin and dexamethasone showed significantly higher gene expression levels of *Gpx3* than those from vehicle-treated or pefloxacin-treated rats (Figure 5D). Further, we investigated the gene expression levels of collagen, the main component of tendon tissue. *Col1a1* was the most abundant subtype of collagen, followed by *Col3a1* (Supplementary Figure S2A). Pefloxacin increased the expression of *Col1a1* and *Col3a1*, indicating a possible compensatory effect against the tendinopathic changes. Dexamethasone decreased their expression levels, and the combination of dexamethasone and pefloxacin downregulated not only the expression levels of *Col1a1* and *Col3a1* but also those of various other collagen subtypes (Supplementary Figure S2B). Furthermore, we examined the expression of inflammation-related genes, reported to be upregulated in tendinopathy (Dakin et al., 2015; Dakin et al., 2018; Abraham et al., 2019; Kendal et al., 2020; Arvind and Huang, 2021). Pefloxacin did not drastically change the expression levels of these genes, indicating that inflammation was not a factor to be considered in this context (Supplementary Figures S2C, D).

Next, we analyzed the expression levels of antioxidant enzymes in human tendons using data from GEO datasets. We found that the expression of *GPX3* was significantly lower in tissues from patients with tendinopathy than in normal tissues, and *GPX3* had a very high expression among all the antioxidant enzymes examined; both findings supported our notion (Supplementary Figure S3). *PRDX4* and *GPX7* were upregulated in tendinopathy samples; however, because their expression levels were quite low, the effect of this upregulation was not assumed to be significant. Therefore, we focused on GPX3 for further analysis.

### Effects of fluoroquinolone and dexamethasone on oxidative stress in rat tenocytes

**FIGURE 6:**
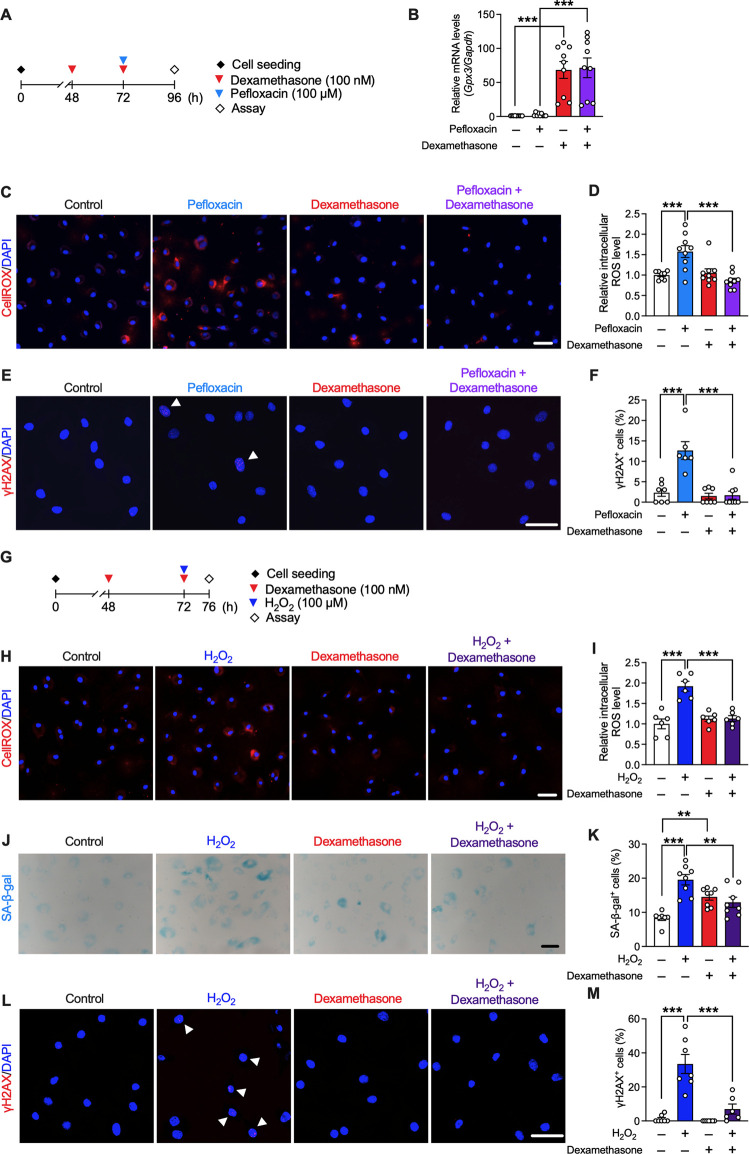
Effects of dexamethasone on pefloxacin-induced toxicity and stress-induced senescence in rat primary tenocytes. **(A)** Primary cultured tenocytes obtained from rat Achilles tendons were treated with pefloxacin (100 µM, 24 h), dexamethasone (100 nM, 24 h pre-treatment plus 24 h co-treatment), or both. **(B)** The gene expression of *Gpx3* after drug treatment was examined using quantitative RT-PCR (*n* = 9 per group) and represented as the relative ratio to the control group. **(C,D)** The levels of reactive oxygen species (ROS) were evaluated using the CellROX assay (*n* = 8–9 per group). Cells were imaged using confocal microscopy, and the intensity of fluorescence was measured in each cell. **(E,F)** The number of cells with three or more γH2AX^+^ foci was counted (shown with an arrowhead) and presented as a percentage of the total cell number. **(G)** Primary cultured tenocytes were treated with H_2_O_2_ (100 µM, 4 h) to create a stress-induced senescence model and co-treated with dexamethasone (100 nM, 24 h pre-treatment plus 4 h co-treatment). **(H,I)** The levels of ROS after drug treatment were evaluated using the CellROX assay (*n* = 6 per group). **(J,K)** Senescence-associated β-galactosidase (SA-β-gal) staining was performed after drug treatment (*n* = 7–8 per group). The number of SA-β-gal^+^ cells was counted and presented as a percentage of the total number of cells. **(L,M)** Immunostaining of γH2AX was performed (*n* = 6–7 per group). The number of cells with three or more γH2AX^+^ foci (shown with arrowhead) is presented as a percentage of the number of total cells. Data are shown as mean ± SEM. Statistical significance was tested using a two-way ANOVA with *post hoc* multiple comparisons; ^**^ *p* < 0.01, ***p < 0.001. Scale bar; 50 µm.

The involvement of oxidative stress and the effect of dexamethasone in fluoroquinolone-induced tendinopathy was validated *in vitro*. Dexamethasone binds to the glucocorticoid receptor and modulates the expression of various genes; hence, we speculated that dexamethasone treatment would result in a preventative effect accompanied by changes in gene expression. Therefore, we conducted pre-treatment with dexamethasone. Primary cultured rat tenocytes were treated with dexamethasone (100 nM, 24 h pre-treatment +24 h treatment), pefloxacin (100 µM, 24 h), or both (Figure 6A). Consistent with the RNA-seq data, dexamethasone drastically upregulated the expression of *Gpx3* (Figure 6B). Tenocytes treated with pefloxacin showed an increase in ROS levels, which was mitigated by co-treatment with dexamethasone (Figures 6C, D). Furthermore, the ratio of γH2AX^+^ cells was elevated by pefloxacin, which was reversed by co-treatment with dexamethasone (Figures 6E, F), consistent with the *in vivo* data (Figures 4F, G).

### Effect of dexamethasone on stress-induced senescence in rat tenocytes

We next examined the effect of dexamethasone on cellular senescence in tenocytes. Oxidative stress is related to several aging conditions, and H_2_O_2_ is used to create a stress-induced senescence model (Frippiat et al., 2000; Wang et al., 2021). Therefore, tenocytes were treated with H_2_O_2_ (100 µM, 4 h) to induce senescence, and dexamethasone (100 nM, 24 h pre-treatment +4 h treatment) was co-administered (Figure 6G). Dexamethasone treatment suppressed the increase in ROS levels induced by H_2_O_2_ (Figures 6H, I). Staining was performed for SA-β-gal and γH2AX; as the level of both SA-β-gal and γH2AX increases during senescence, these are used as biomarkers of senescence (Wan et al., 2021). Although dexamethasone treatment slightly increased the ratio of SA-β-gal^+^ cells, H_2_O_2_-induced cellular senescence was suppressed by co-treatment with dexamethasone (Figures 6J–M).

### Effect of GPX3 on fluoroquinolone-induced oxidative stress in rat tenocytes

**FIGURE 7:**
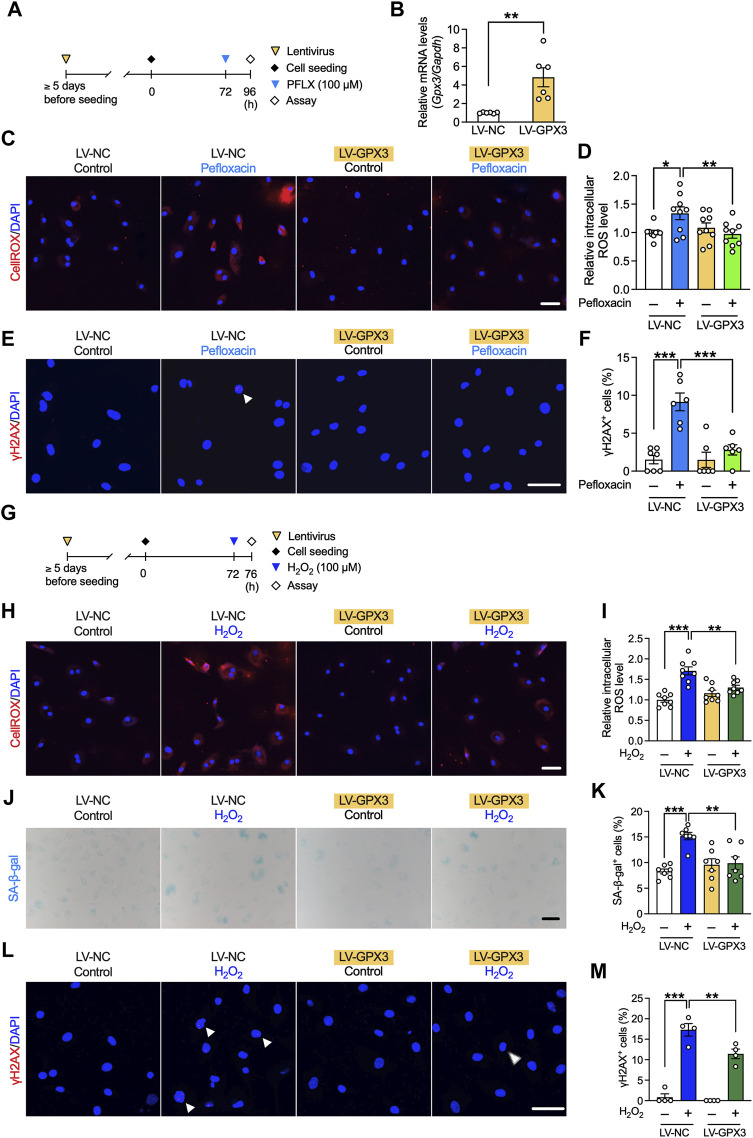
Effects of GPX3 on pefloxacin-induced oxidative stress and hydrogen peroxide-induced senescence in rat primary tenocytes. **(A)** Tenocytes transduced with lentivirus for overexpressing GPX3 (LV-GPX3) or negative control (LV-NC). **(B)** The gene expression of *Gpx3* after lentiviral induction was examined using quantitative RT-PCR (*n* = 6 per group) and represented as the relative ratio to the LV-NC group. **(C,D)** The levels of reactive oxygen species (ROS) were evaluated using the CellROX assay (*n* = 9 per group), and cells were imaged using confocal microscopy. The intensity of fluorescence was measured in each cell. **(E,F)** Tenocytes were stained with anti-γH2AX antibodies (*n* = 6–7 per group). The number of cells with three or more γH2AX^+^ foci (shown with arrowhead) is presented as a percentage of the total number of cells. **(G)** To create a stress-induced senescence model, tenocytes were treated with H_2_O_2_ (100 µM, 4 h). **(H,I)** The levels of ROS were evaluated using the CellROX assay (*n* = 8 per group). **(J,K)** SA-β-gal staining was performed (*n* = 7 per group); the number of SA-β-gal^+^ cells are presented as a percentage of the number of total cells. **(L,M)** Cells were stained with anti-γH2AX antibody (*n* = 4 per group). The number of cells with three or more γH2AX^+^ foci (shown with arrowhead) is presented as a percentage of the total number of cells. Data are shown as mean ± SEM. Statistical significance was tested using a two-way ANOVA with *post hoc* multiple comparisons; *p < 0.05, **p < 0.01, ***p < 0.001. Scale bar; 50 µm.

To investigate whether the observed effects of dexamethasone occurred mainly through the upregulation of GPX3, we next examined the role of GPX3. Lentivirus was transfected into tenocytes to induce GPX3 overexpression, and pefloxacin (100 µM, 24 h) was administered (Figure 7A). The expression level of *Gpx3* significantly increased in GPX3-overexpressing tenocytes (Figure 7B), whereas that of the other antioxidant enzymes or collagen type 1 was unaltered (Supplementary Figure S4). Overexpression of GPX3 suppressed the increase in ROS levels and the ratio of γH2AX^+^ cells caused by pefloxacin (Figures 7C–F).

### Effect of GPX3 on stress-induced senescence model in rat tenocytes

The role of GPX3 in cellular senescence was also investigated using the stress-induced senescence model created *via* treatment with H_2_O_2_ (Figure 7G). The increased ROS levels due to H_2_O_2_ treatment were suppressed by the overexpression of GPX3, and this effect was not as strong as that by dexamethasone; however, it was correlated with the expression level of GPX3 (Figures 7H, I). The increase in the number of SA-β-gal^+^ and γH2AX^+^ cells was partly suppressed by the overexpression of GPX3 (Figures 7J–M).

## Discussion

To the best of our knowledge, this is the first study to report that glucocorticoid dexamethasone can mitigate fluoroquinolone-induced and age-related tendinopathy in humans. These findings were observed in our analysis of four independent large clinical datasets, and the hypothesis was further validated *via in vivo* and *in vitro* studies on rats. Dexamethasone treatment suppressed oxidative stress related to fluoroquinolone-induced and age-related tendinopathy by upregulating the expression of the antioxidant enzyme GPX3.

Our data mining of the FAERS, JADER, and CVARD data showed that fluoroquinolone use resulted in specific signals of tendinopathy, with high ROR values in thousands of cases across all three databases. These signals were considered sufficiently robust because they were based on large datasets, irrespective of the diversity of the clinical circumstances. We focused on the effect of dexamethasone because it was the only common drug with a significantly low ROR value among all three databases.

The resultant hypothesis after the analysis of data from these spontaneous reporting systems was further validated in a retrospective cohort analysis using the IBM MarketScan data. The results revealed that dexamethasone treatment mitigated the risk of developing fluoroquinolone-induced tendinopathy. Moreover, dexamethasone alleviated the risk of age-related tendinopathy. The suppressive effect of dexamethasone was less strong in age-related tendinopathy than in fluoroquinolone-induced tendinopathy, which may be due to the age difference. Tendons are consistently under mechanical stress in daily life, and repetitive strain can lead to the accumulation of microdamage, increasing the risk of developing tendinopathy (Wu et al., 2017). The healing ability of tendons decreases with age (Zhou et al., 2014); hence, there may be more microtears in the tendons of older adults than those of younger adults. In our analysis, the median age of the fluoroquinolone cohort was 51–52 years, whereas the median age of the cohort of older adults was 73 years. Owing to this large age gap, the beneficial effect of dexamethasone may have been partly masked in the older adult cohort.

However, several previous retrospective analyses have shown that glucocorticoid use increases the risk of tendinopathy (Wong et al., 2003; Zhang et al., 2013; Ge et al., 2020), and FDA has warned of a high risk of developing fluoroquinolone-induced tendinopathy in those consuming steroids (Tanne, 2008). This discrepancy may be due to the length of the dexamethasone administration period. In a previous clinical study, the Achilles tendons of patients with rheumatoid arthritis who received dexamethasone treatment for at least 3 months had a low density of collagen type 1 (Abraham et al., 2019). *In vitro* analyses have shown that dexamethasone treatment reduces the gene expression of collagen type 1, collagen synthesis, and proliferation of tenocytes (Wong et al., 2003; Zhang et al., 2013). Further, our RNA-seq analysis of the Achilles tendons from drug-administered rats revealed that dexamethasone downregulated collagen subtypes with high expression in tendon tissues. Therefore, long-term administration of dexamethasone may harm tendons by reducing collagen production. Nonetheless, the effect of its short-term treatment remains unclear. A previous case-control study showed that continuous glucocorticoid treatment increased the odds ratio of tendinopathy, whereas intermittent use exhibited low odds ratio (Spoendlin et al., 2015). In a recent clinical trial, local injections of steroids (maximum three times at 4-week intervals) combined with exercise showed better outcomes in the treatment of tendinopathy than with placebo injections and exercise at 6 months (Johannsen et al., 2022). In the rat tendon injury model, tendon healing was enhanced, and their mechanical properties improved when dexamethasone was administered for 5 days (Dietrich-Zagonel et al., 2022). In our analysis of fluoroquinolone-induced and age-related tendinopathy from the IBM MarketScan data, the median administration period of dexamethasone was 12 and 10 days in the fluoroquinolone and older adult cohort, respectively. In our *in vivo* study, dexamethasone alone did not influence the maximum stress of Achilles tendons after 15 times of its administration. Because the turnover of tendons is slow (Heinemeier et al., 2013), the negative effect of dexamethasone did not appear at the time of the investigation. Whether dexamethasone is beneficial or disadvantageous to tendons may rely on its administration duration.

Accumulating evidence has indicated the involvement of oxidative stress in tendinopathy. In previous studies, ROS levels were increased in patients with tendinopathy (Yoshida et al., 2020), and an *in vivo* study showed that tendinopathic changes in the patellar tendon are attributed to H_2_O_2_ (Fu et al., 2018). Fluoroquinolones cause oxidative stress in tenocytes (Pouzaud et al., 2004; Lowes et al., 2009). According to a previous report, fluoroquinolones generated ROS in human tenocytes through mitochondrial membrane damage, which was mitigated by MitoQ, a mitochondria-targeted antioxidant (Lowes et al., 2009). Furthermore, patients taking fluoroquinolones showed an increase in lipid peroxide and a decrease in plasma antioxidant contents, both suggesting an increase in ROS levels (Talla and Veerareddy, 2011). Oxidative stress is also related to several age-related conditions, and an age-related increase in ROS production and oxidative stress has been reported in various tissues, including tendons (Cadenas and Davies, 2000; Radák et al., 2002). The production of reactive carbonyl derivatives, a well-known biomarker for oxidative damage, significantly increases in aged Achilles tendons (Radák et al., 2002). Consistent with previous findings, our gene ontology analysis of the gene expression in Achilles tendons from drug-administered rats revealed that oxidative stress was involved in fluoroquinolone-induced tendinopathy. Aging-related pathways were also enriched, possibly because fluoroquinolones and aging are related to oxidative stress. ROS, related to the increased expression of matrix metalloproteinases (Wunderli et al., 2020), activate several pathways, including the c-Jun N-terminal kinase (JNK) (Wang et al., 2007) and NF-κB pathways (Best et al., 2020); however, further studies are required to elucidate the manner in which oxidative stress leads to tendinopathy.

We found that dexamethasone mitigated fluoroquinolone-induced oxidative stress primarily through the upregulation of GPX3, an antioxidant belonging to the glutathione peroxidase family. GPX3 is a selenoprotein containing selenocysteine in its active site. Its major role is to eliminate H_2_O_2_, hydroperoxides, and lipid hydroperoxides by catalyzing the conversion of glutathione to oxidized glutathione (Chang et al., 2020). GPX3 is mostly secreted and therefore abundant in plasma; additionally, it is present intracellularly in various tissues, including the kidney, heart, lung, and liver (Nirgude and Choudhary, 2021). The promoter region of the *GPX3* gene contains glucocorticoid-binding elements (GRE). Therefore, the substantial upregulation of GPX3 by dexamethasone observed in our *in vitro* study may be mediated by GRE. Additionally, a binding site for hypoxia-inducible factor-1 (HIF-1) is present in the promoter region. Because tendons are under low O_2_ pressure because of hypovascularity, we speculate that HIF-1 is activated constantly, leading to the upregulation of GPX3. Our *in vivo* study and GEO data analysis showed that the gene expression of *GPX3* was relatively high among antioxidant enzymes in both rat Achilles and human tendons. Although several studies have been conducted on other musculoskeletal tissues, only a few have focused on GPX3 in tendons. In human myocytes, GPX3 plays a crucial role in maintaining cell viability (El Haddad et al., 2012). Furthermore, methylation in the promoter region of GPX3 and downregulation of *GPX3* have been observed in patients with Kashin–Beck disease, an endemic osteoarthropathy (Han et al., 2018). Further studies are required to determine the role of GPX3 in tendons and tendinopathy.

Our *in vitro* study showed that dexamethasone treatment also suppressed the cellular senescence induced by H_2_O_2_. However, a slight increase in the number of SA-β-gal^+^ cells was observed after dexamethasone treatment. In a previous study, dexamethasone treatment increased the number of SA-β-gal^+^ tenocytes when treated for 3 days (Poulsen et al., 2014). We did not observe an increase in ROS levels or the number of γH2AX^+^ cells by dexamethasone treatment; hence, we speculated that dexamethasone could have increased the number of SA-β-gal^+^ cells through pathways other than oxidative stress. Examining cellular senescence in GPX3-overexpressing tenocytes revealed that GPX3 mitigated cellular senescence. The expression of GPX3 reportedly declines with age (Pastori et al., 2016), possibly increasing ROS levels in older adults. In our *in vitro* study, the effect of GPX3 overexpression on H_2_O_2_-induced cellular senescence was not as strong as that of dexamethasone; nevertheless, it correlated with the expression of GPX3. The optimum expression of GPX3 required for preventing tendinopathy needs to be investigated further.

Our study has some limitations. First, the role of GPX3 was not validated in the rat model. We were not able to inject lentivirus to rat Achilles tendon due to practical issue of injection. Overexpression and/or knockdown of GPX3 in tendon tissues by lentivirus or examination of tendons from *Gpx3* knockout mice may provide valuable information regarding the effect of GPX3 on tendinopathy. Second, the protein levels of GPX3 in drug-treated tenocytes or rat Achilles tendons were not investigated. GPX3 is a selenoprotein, and its translation is highly sensitive to the level of selenium (Chang et al., 2020). The level of selenite in tendons is unknown, and the activity of GPX in chondrocytes reportedly increases by adding sodium selenite (50 nM) for 24 h (Yan et al., 2013). Hence, we added sodium selenite (100 nM) in the media, whose concentration was presumably sufficient for the translation of GPX3, when it was changed to serum-free media to conduct drug treatment. Future studies should include a way to measure the protein level or activity of GPX3. Third, we did not investigate the cell cycle or apoptosis in the oxidative stress-induced senescence model. A stable cell cycle arrest and resistance to apoptosis are other phenotypes of cellular senescence. The proliferation rate of tenocytes is slow, requiring more than 1 week as a doubling time. Therefore, exploring the cell cycle was not suitable for our administration schedule, where H_2_O_2_ was treated for only 4 h. However, these phenotypes will be examined in the future for an in-depth analysis.

**FIGURE 8:**
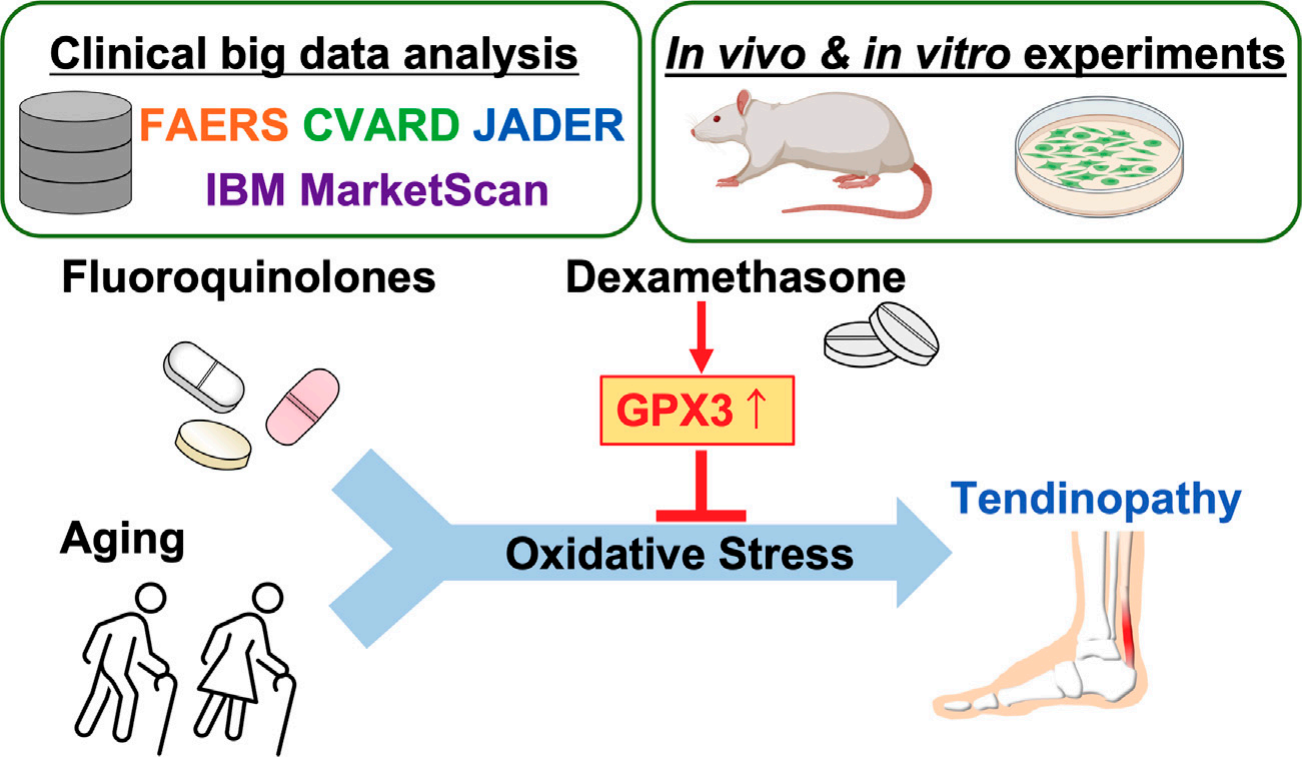
Graphical summary of the study. Created with BioRender.com.

In conclusion, our findings demonstrated that dexamethasone treatment effectively reduced tendinopathy in both human retrospective analyses and experimental models. Nonetheless, the long-term use of dexamethasone may have hazardous effects on the tendon, including a reduction in the content of collagen and other well-known side effects, such as osteoporosis and sleep disturbance. Thus, a novel molecule that selectively upregulates or activates GPX3 needs to be identified for treatment (Figure 8).

## Data Availability

The datasets presented in this study can be found in online repositories. The names of the repository/repositories and accession number(s) can be found in the article/Supplementary Material.
