# Supplementary material for: Increased expression of glutathione peroxidase 3 prevents tendinopathy by suppressing oxidative stress
Source: Front Pharmacol. 2023 Mar 20;14:1137952. doi: 10.3389/fphar.2023.1137952 (PMC10067742; doi:10.3389/fphar.2023.1137952)
Supplement: Supplementary file 1 [file DataSheet1.docx]

Supplementary Material

Increased expression of glutathione peroxidase 3 prevents tendinopathy by suppressing oxidative stress

Haruka Furuta^1^, Mari Yamada^1^, Takuya Nagashima^1^, Shuichi Matsuda^2^, Kazuki Nagayasu^1^, Hisashi Shirakawa^1^, Shuji Kaneko^1*^

*** Correspondence:** Shuji Kaneko: skaneko@pharm.kyoto-u.ac.jp

# Supplementary Materials

**
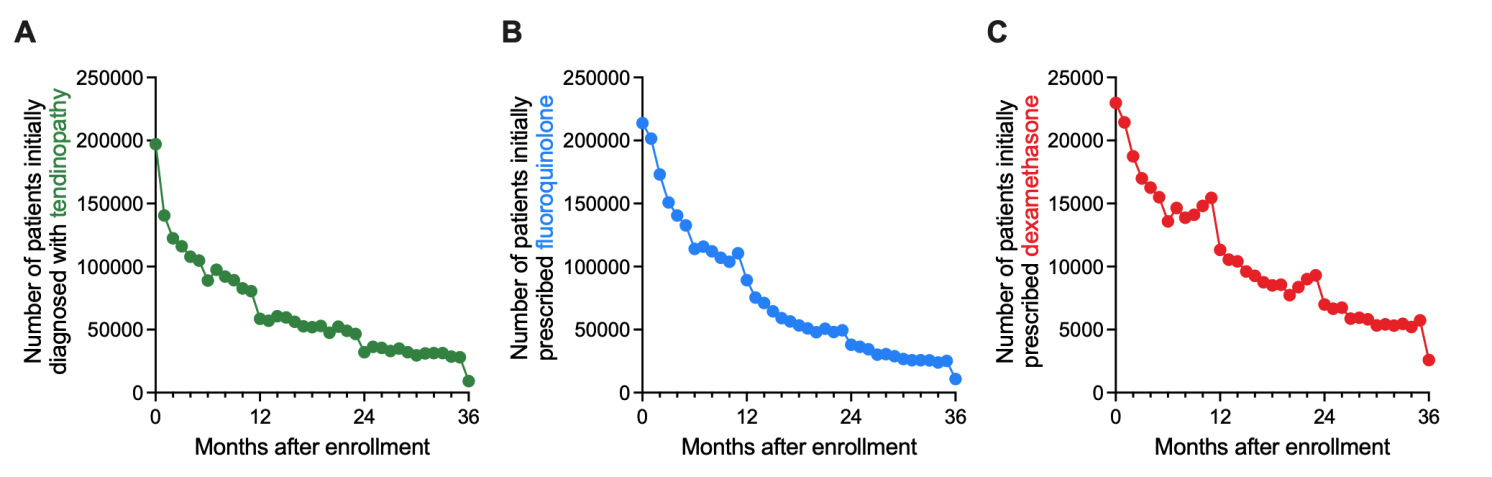
**

**Figure S1. Intervals of insurance enrollment obtained from IBM MarketScan data.** Intervals of insurance enrollment of a patient who was first diagnosed with tendinopathy (A) and first prescription of fluoroquinolone (B) and dexamethasone (C) were analyzed; the number of patients is on a monthly basis. From these data, data for patients who had been enrolled for health insurance for 0–5 months were excluded from the analysis to allow for a run-in period.


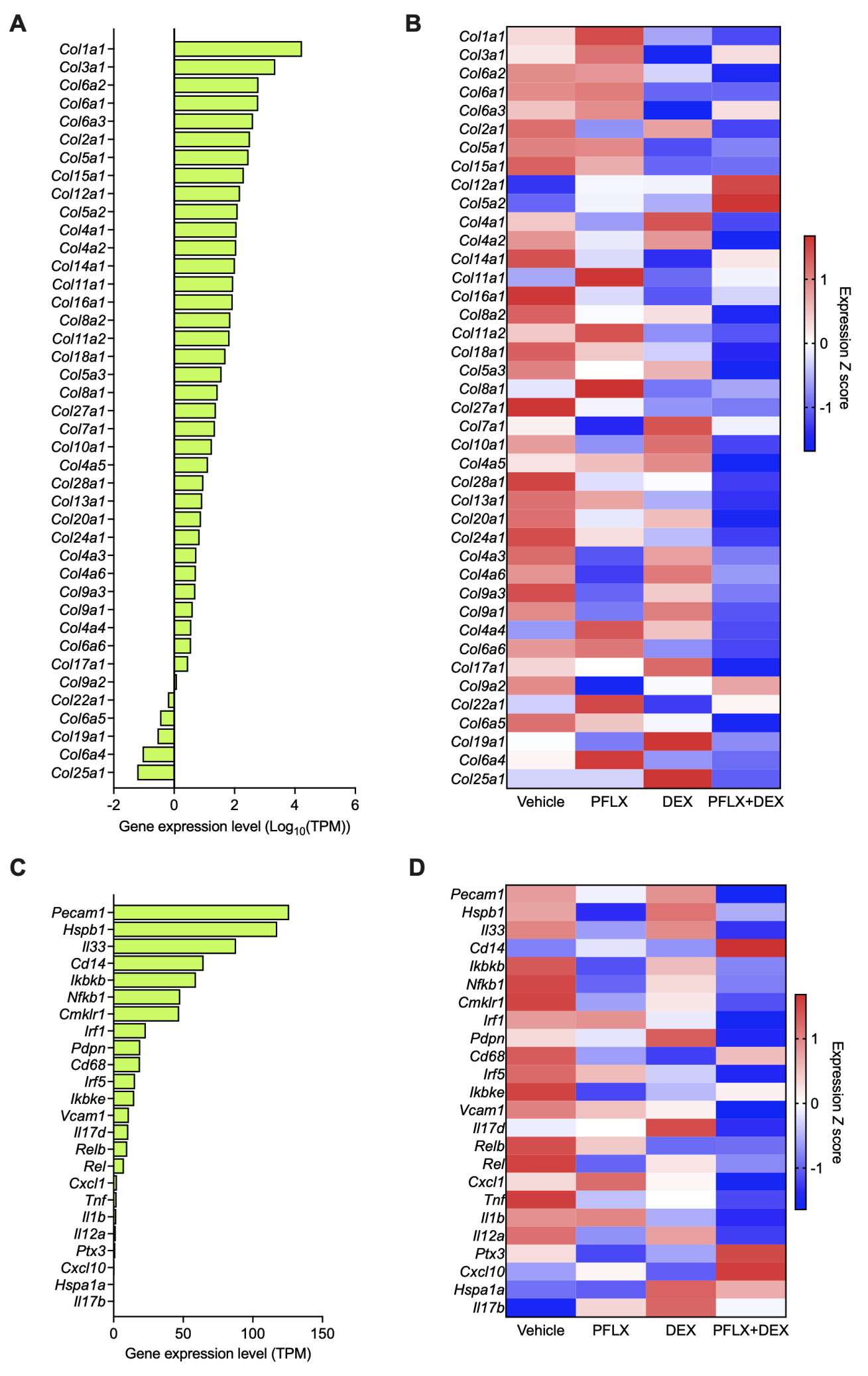


**Figure S2. Gene expression in tendon tissues of rats treated with pefloxacin, dexamethasone, or both.** (**A**) The relative gene expression levels, shown as the common logarithm of transcripts per million (TPM), of collagens in the Achilles tendon from a vehicle-administered rat. (**B**) Heatmap of gene expression of collagens in Achilles tendon from rats administered vehicle, pefloxacin (PFLX), dexamethasone (DEX), or both (PFLX+DEX). (**C**) The relative gene expression levels, shown as TPM, of inflammation-related genes in the Achilles tendon from a vehicle-administered rat. (**D**) Heatmap of the expression of inflammation-related genes in Achilles tendon from rats administered vehicle, pefloxacin (PFLX), dexamethasone (DEX), or both (PFLX+DEX). In the heatmaps, red and blue colors represent high and low gene expression levels, respectively.


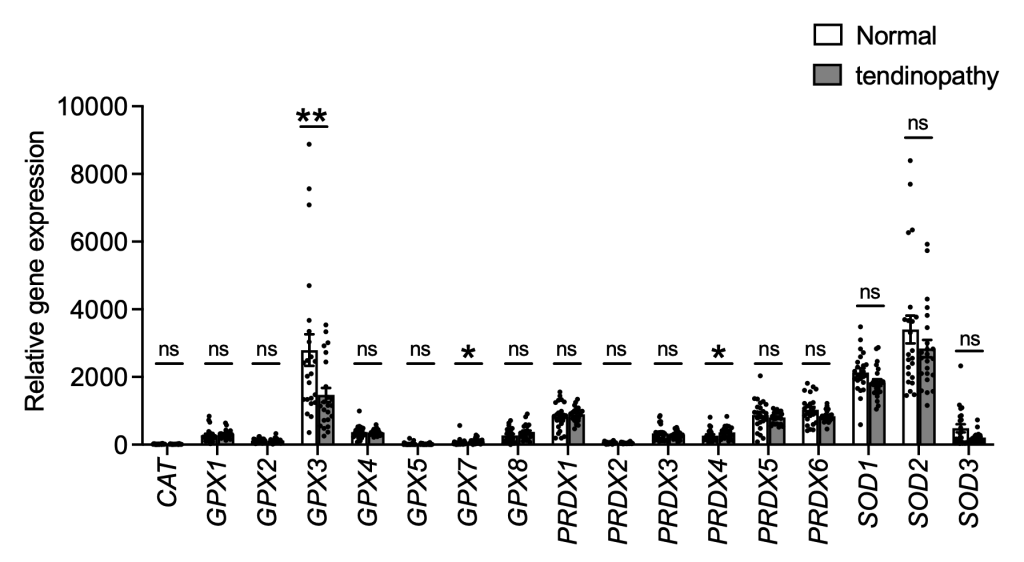


**Figure S3. Expression of antioxidant enzymes in human tendinopathy.** The expression levels of antioxidant enzymes in gene expression omnibus (GEO) datasets obtained from human tendinopathy and normal tendon tissues (*n* = 23). Data are shown as the mean ± SEM. Statistical significance in each gene was tested using a ratio paired *t*-test; **p* < 0.05, ***p* < 0.01, ns: not significant.


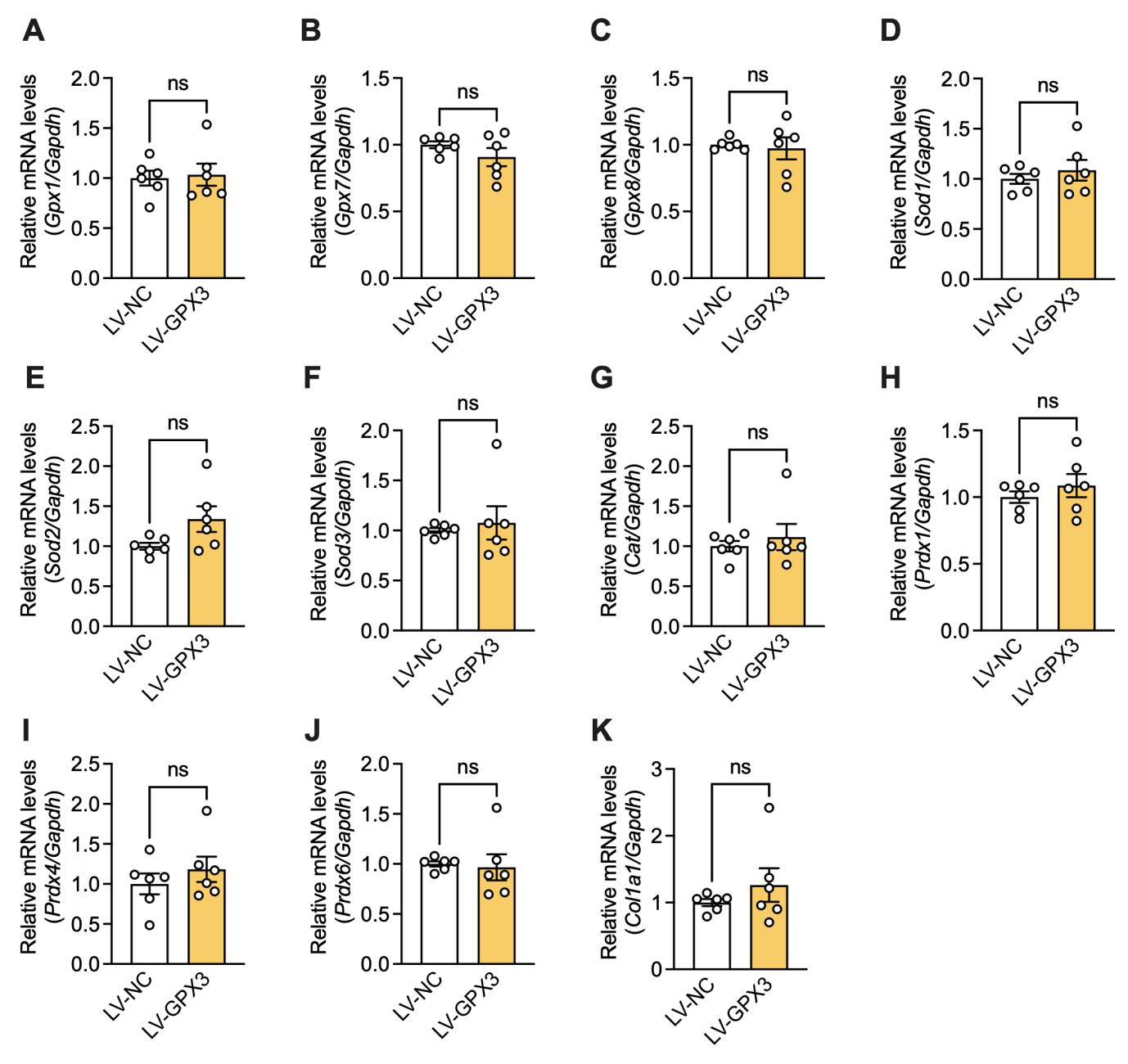


**Figure S4. Effect of GPX3 overexpression on the gene expression of other antioxidant enzymes and collagen type 1.** Rat primary tenocytes were transfected with lentivirus for the overexpression of GPX3 (LV-GPX3) or negative control (LV-NC), as shown in Figure 7A. The gene expression levels were examined using quantitative RT-PCR. Expression levels of *Gpx1* (A), *Gpx7* (B), *Gpx8* (C), *Sod1* (D), *Sod2* (E), *Sod3* (F), *Cat* (G), *Prdx1* (H), *Prdx4* (I), *Prdx6* (J), and *Col1a1* (K) were normalized to that of *Gapdh* in each sample (n = 6 per group). Data are shown as the mean ± SEM. Statistical significance in each gene was tested using Student’s *t*-test; ns: not significant.

**Data S1. Comprehensive results of the disproportionality analysis for tendinopathy in the FDA Adverse Event Reporting System (FAERS), Canada Vigilance Adverse Reaction Online Database (CVARD), and Japanese Adverse Drug Event Report (JADER) data.**

Filename: DataS1_FAERS_CVARD_JADER_DrugA.xlsx (Three separate tab sheets)

Individuals in the database were divided into four groups as follows: (*a*) individuals who received the drug of interest (drug A) and exhibited tendinopathy, (*b*) individuals who received Drug A but did not exhibit tendinopathy, (*c*) individuals who did not receive Drug A and exhibited tendinopathy, and (*d*) individuals who did not receive Drug A and did not exhibit tendinopathy. To include Drug A with either (*a*), (*b*), (*c*), or (*d*) equal to 0, Haldane–Anscombe correction was applied. The reporting odds ratio (ROR) with a 95% confidence interval (CI) and *Z-*score were calculated using the following equations:

ROR = $\frac{\frac{a}{b}}{\frac{c}{d}}$

95% CI = exp$\left\{ log(\mathrm{ROR})\pm1.96\sqrt{\frac{1}{a}+\frac{1}{b}+\frac{1}{c}+\frac{1}{d}} \right\}$

*Z* score = $\frac{log(\mathrm{ROR})}{\sqrt{\frac{1}{a}+\frac{1}{b}+\frac{1}{c}+\frac{1}{d}}}$

where *a*, *b*, *c*, and *d* refer to the number of individuals in each group, adjusted using the Haldane–Anscombe correction.

**Data S2. Comprehensive confounding effects of the concomitant drug (Drug B) on tendinopathy associated with fluoroquinolone in the FDA Adverse Event Reporting System (FAERS), Canada Vigilance Adverse Reaction Online Database (CVARD), and Japanese Adverse Drug Event Report (JADER) data.**

Filename: DataS2_FAERS_CVARD_JADER_DrugB.xlsx (three separate tab sheets)

Individuals who received fluoroquinolone were divided into four groups as follows: (*a1*) individuals who received the concomitant drug of interest (Drug B) and exhibited tendinopathy, (*b1*) individuals who received Drug B but did not exhibit tendinopathy, (*c1*) individuals who did not receive Drug B and exhibited tendinopathy, and (*d1*) individuals who did not receive Drug B and did not exhibit tendinopathy. The Haldane–Anscombe correction was applied to the analyses. The reporting odds ratio (ROR) with a 95% confidence interval (CI) and *Z* score for fluoroquinolone-induced tendinopathy were calculated using the following equations:

ROR = $\frac{\frac{a1}{b1}}{\frac{c1}{d1}}$

95% CI = exp$\left\{ log(\mathrm{ROR})\pm1.96\sqrt{\frac{1}{a1}+\frac{1}{b1}+\frac{1}{c1}+\frac{1}{d1}} \right\}$

*Z* score = $\frac{log(\mathrm{ROR})}{\sqrt{\frac{1}{a1}+\frac{1}{b1}+\frac{1}{c1}+\frac{1}{d1}}}$

where a1, b1, c1, and d1 refer to the number of individuals in each group, adjusted using the Haldane–Anscombe correction.

**Table S1. Definition of diseases and drugs in the IBM MarketScan data**

| Diseases | |
| --- | --- |
| Tendinopathy | M654, M658, M659, M662, M663, M668, M669, M751, M752, M754, M760, M761, M765, M766, M767, M768, M769, M771, M775, M778, M779, S860 |
| Renal failure | N17, N18, N19 |
| Obesity | E65, E66 |
| Diabetes | E10, E11, E12, E13, E14 |
| Rheumatoid arthritis | M05, M06 |
| Gout | M10 |
| Cancer | C00, C01, C02, C03, C04M, C05, C06, C07, C08, C09, C10, C11, C12, C13, C14, C15, C16, C17, C18, C19, C20, C21, C22, C23, C24, C25, C26, C30, C31, C32, C33, C34, C37, C38, C39, C40, C41, C43, C44, C45, C46, C47, C48, C49, C4A, C50, C51, C52, C53, C54, C55, C56, C57, C58, C60, C61, C62, C63, C64, C65, C66, C67, C68, C69, C70, C71, C72, C73, C74, C75, C76, C77, C78, C79, C7A, C7B, C80, C81, C82, C83, C84, C85, C86, C88, C90, C91, C92, C93, C94, C95, C96, D00, D01, D02, D03, D04, D05, D06, D07, D09 |
| Liver disease | K70, K71, K72, K73, K74, K75, K76, K77 |
| Hyperthyroidism | E05 |
| Musculoskeletal disease | M00, M01, M02, M04, M05, M06, M07, M08, M10, M11, M12, M13, M14, M15, M16, M17, M18, M19, M1A, M20, M21, M22, M23, M24, M25, M26, M27, M30, M31, M32, M33, M34, M35, M36, M40, M41, M42, M43, M45, M46, M47, M48, M49, M50, M51, M53, M54, M60, M61, M62, M63, M67, M70, M71, M72, M79, M80, M81, M83, M84, M85, M86, M87, M88, M89, M90, M91, M92, M93, M94, M95, M96, M97, M99, M650, M651, M652, M653, M660, M661, M753, M762, M763, M764, M772, M773, M774 |
| Heart disease | I05, I06, I07, I08, I09, I20, I21, I22, I23, I24, I25, I26, I27, I28, I34, I35, I36, I37, I38, I39, I42, I43, I44, I45, I47, I48, I49, I50, I51, I52, Q20, Q21, Q22, Q23, Q24 |
| Lipid disorder | E38 |
| Drugs | |
| Fluoroquinolone | Alatrofloxacin Mesylate, Ciprofloxacin, Ciprofloxacin Hydrochloride,Ciprofloxacin/Ciprofloxacin Hydrochloride, Delafloxacin, Dextrose/Levofloxacin, Enoxacin, Gatifloxacin, Gemifloxacin Mesylate, Grepafloxacin Hydrochloride, Levofloxacin, Lomefloxacin Hydrochloride, Moxifloxacin Hydrochloride, Norfloxacin, Ofloxacin, Sparfloxacin, Trovafloxacin Mesylate |
| Dexamethasone | Dexamethasone, Dexamethasone Acetate, Dexamethasone Sodium Phosphate, Dexamethasone Sodium Phosphate/Dextrose, Dexamethasone Sodium Phosphate/Sodium Chloride |
| Statin | Amlodipine Besylate/Atorvastatin Calcium, Aspirin;Pravastatin Sodium, Atorvastatin Calcium, Atorvastatin Calcium/Ezetimibe, Cerivastatin Sodium, Ezetimibe/Simvastatin, Fluvastatin Sodium, Lovastatin, Lovastatin/Niacin, Niacin/Simvastatin, Pitavastatin, Pitavastatin Calcium, Pravastatin Sodium, Rosuvastatin Calcium, Simvastatin, Simvastatin/Sitagliptin Phosphate |
| Aromatase inhibitor | Aminoglutethimide, Anastrozole, Exemestane, Letrozole, Letrozole;Ribociclib, Testolactone |

**Table S2**. **Oligodeoxynucleotide primer list used for quantitative RT-PCR**

| For quantitative RT-PCR | |
| --- | --- |
| *Gapdh* | 5'-GTG CCA GCC TCG TCT CAT AG-3' |
|  | 5'-AGA GAA GGC AGC CCT GGT AA-3' |
| *Gpx1* | 5'-AGT TCG GAC ATC AGG AGA ATG G-3' |
|  | 5'-AAA GAG CGG GTG AGC CTT C-3' |
| *Gpx3* | 5'-ATT CGG CCT GGT CAT TCT GG-3' |
|  | 5'-CCC GGT CGA ACG TAC TTG AG-3' |
| *Gpx7* | 5'-AAG TAC CGC GGC TCG GTT T-3' |
|  | 5'-TGC AAG GCT CGG TAG TTC TG-3' |
| *Gpx8* | 5'-TGA AGG ATG CCA AAG GAA GGA-3' |
|  | 5'-ACG TTT ACA ACT AGG GAA GCT TTG-3' |
| *Sod1* | 5'-CTT CTG TCG TCT CCT TGC TT-3' |
|  | 5'-TTC ACC GCT TGC CCT TCT G-3' |
| *Sod2* | 5'-GGA GCA AGG TCG CTT ACA GA-3' |
|  | 5'-AGC AGT GGA ATA AGG CCT GTG-3' |
| *Sod3* | 5'-GCT GGG TCT GTC CTG TAC TT-3' |
|  | 5'-CTG AGG TTC CAC ACC TGA CAA G-3' |
| *Cat* | 5'-ATG GCT ATG GCT CAC ACA CC-3' |
|  | 5'-TTG ATG CCC TGG TCA GTC TTG-3' |
| *Prdx1* | 5'-CTG TAG CTC GAC TCT GCT GA T-3' |
|  | 5'-TTG AAG CTG GGA GCA GGA TG-3' |
| *Prdx4* | 5'-TGA GAC ACT GCG TTT GGT TC-3' |
|  | 5'-GTT TCA CTA CCA GGT TTC CAG-3' |
| *Prdx6* | 5'-TGC CTG GAG CAA GGA CAT CAA-3' |
|  | 5'-CCC AAC AGG ATG GCA AGG TC-3' |
| *Col1a1* | 5'-GTG CCA GCC TCG TCT CAT AG-3' |
|  | 5'-AGA GAA GGC AGC CCT GGT AA-3' |
| *Actb* | 5’-GTC CAC CCG CGA GTA CAA C-3’ |
|  | 5’-TAT CGT CAT CCA TGG CGA ACT GG-3’ |
| For vector construction | |
| *Cag* | 5'-AAT GCT AGC TAT TAA TAG TAA TCA ATT AC-3' |
|  | 5'-ATT GCG GCC GCT TTG CCA AAA TGA TGA GAC A-3' |
| *Gpx3* | 5'-AAT GCG GCC GCA TGG CCC GGA TCC TTC GGG C-3' |
|  | 5'-TTA GAA TTC TTA CTT CCC TCT GGC CCC CA-3' |

**Table S3. Reporting odds ratio (ROR) and *Z* score of the concomitant drugs on tendinopathy associated with fluoroquinolone with *Z* score <−1.96 in the FDA Adverse Event Reporting System (FAERS), Canada Vigilance Adverse Reaction Online Database (CVARD), and Japanese Adverse Drug Event Report (JADER) data.** ROR and 95% confidence interval (CI) are shown.

|  | **FAERS** | | **CVARD** | | **JADER** | |
| --- | --- | --- | --- | --- | --- | --- |
|  | ROR  (95% CI) | *Z* score | ROR  (95% CI) | *Z* score | ROR  (95% CI) | *Z* score |
| Dexamethasone | 0.040  (0.024–0.066) | −12.61 | 0.061  (0.004–0.978) | −1.98 | 0.118  (0.023–0.588) | −2.61 |
| Vancomycin | 0.061  (0.043–0.088) | −15.07 | 0.026  (0.002–0.425) | −2.56 | 0.244  (0.070–0.851) | −2.21 |
| Piperacillin | 0.086  (0.063–0.117) | −15.46 | 0.044  (0.003–0.709) | −2.20 | 0.237  (0.068–0.827) | −2.26 |
| Sulfamethoxazole | 0.081  (0.061–0.106) | −18.05 | 0.307  (0.120–0.788) | −2.46 | 0.105  (0.030–0.366) | −3.54 |
| Furosemide | 0.124  (0.105–0.146) | −24.87 | 0.413  (0.270–0.630) | −4.10 | 0.402  (0.202–0.802) | −2.59 |
| Trimethoprim | 0.148  (0.121–0.181) | −18.60 | 0.302  (0.118–0.775) | −2.49 | 0.105  (0.030–0.367) | −3.53 |
| Acetaminophen | 0.290  (0.264–0.319) | −25.71 | 0.425  (0.303–0.595) | −4.98 | 0.278  (0.127–0.609) | −3.20 |

**Table S4. Reporting odds ratio (ROR) and *Z* score of concomitant glucocorticoids except for dexamethasone on tendinopathy associated with fluoroquinolone in the FDA Adverse Event Reporting System (FAERS), Canada Vigilance Adverse Reaction Online Database (CVARD), and Japanese Adverse Drug Event Report (JADER) data.** ROR and 95% confidence interval (CI) are shown.

|  | **FAERS** | | **CVARD** | | **JADER** | |
| --- | --- | --- | --- | --- | --- | --- |
|  | ROR  (95% CI) | *Z* score | ROR  (95% CI) | *Z* score | ROR  (95% CI) | *Z* score |
| Methylprednisolone | 0.38  (0.31–0.47) | −9.06 | 0.26  (0.09–0.75) | −2.49 | 0.95  (0.45–1.97) | −0.15 |
| Prednisolone | 0.49  (0.41–0.59) | −7.65 | 4.93  (1.14–21.3) | 2.14 | 1.78  (1.21–2.63) | 2.94 |
| Prednisone | 0.61  (0.53–0.70) | −6.94 | 0.58  (0.35–0.96) | −2.11 | 104.2  (2.06–5267) | 2.32 |
| Hydrocortisone | 0.11  (0.05–0.20) | −6.88 | 0.06  (0.003–0.96) | −1.99 | 0.47  (0.14–1.65) | −1.17 |
| Betamethasone | 0.23  (0.10–0.53) | ­−3.42 | 1.42  (0.08–26.4) | 0.23 | 0.37  (0.07–1.86) | −1.20 |
| Triamcinolone | 0.45  (0.15–1.32) | −1.46 | 12.8  (0.25–645) | 1.27 | 3.35  (0.20–56.3) | 0.84 |
| Deflazacort | 0.22  (0.01–3.69) | −1.05 | 12.8  (0.25–645) | 1.27 | 104.2  (2.06–5267) | 2.32 |
| Meprednisone | 0.66  (0.04–12.2) | −0.28 | 12.8  (0.25–645) | 1.27 | 104.2  (2.06–5267) | 2.32 |
| Cortisone | 0.88  (0.29–2.71) | −0.22 | 0.75  (0.04–13.0) | −0.20 | 34.7  (1.41–856) | 2.17 |
| Fluocortolone | 5.92  (0.11–298) | 0.89 | 12.8  (0.25–645) | 1.27 | 104.2  (2.06–5267) | 2.32 |

**Table S5**. **Patient backgrounds, diagnoses accompanied with dexamethasone, daily and cumulative doses, and details of tendinopathy in the original and matched cohorts who were prescribed fluoroquinolones.** (a) The number of patients for each confounding factor. (b) The number of patients and their percentage with respect to the total number of patients in each ICD-10 class. Top 10 ICD-10 classes by percentage with more than 10 patients are listed. (c) The defined daily dose (DDD) was used to calculate the daily dose of fluoroquinolones. The median value and interquartile range (IQR) for each group. (d) The number of patients and their proportion with respect to the total number in the cohort.

| (a) |  | | |  | | |
| --- | --- | --- | --- | --- | --- | --- |
| Population with fluoroquinolone | Before matching | | | After matching | | |
|  | Without  dexamethasone | With  dexamethasone | *p* value | Without dexamethasone | With  dexamethasone | *p* value |
| Total | 1,562,000 | 5,063 | – | 5,063 | 5,063 | – |
| Median age (IQR) | 49 (36–59) | 51 (39–59) | <0.0001 | 52 (39–59) | 51 (39–59) | 0.42 |
| Female | 963,807 | 3,175 | 0.14 | 3,170 | 3,175 | 0.92 |
| Renal Failure | 97,810 | 463 | <0.0001 | 445 | 463 | 0.53 |
| Obesity | 365,025 | 1,242 | 0.051 | 1,227 | 1,242 | 0.73 |
| Diabetes | 270,264 | 900 | 0.37 | 914 | 900 | 0.72 |
| Rheumatoid arthritis | 34,860 | 117 | 0.70 | 114 | 117 | 0.84 |
| Gout | 39,367 | 125 | 0.82 | 118 | 125 | 0.65 |
| Cancer | 196,670 | 2,484 | <0.0001 | 2,493 | 2,484 | 0.86 |
| Liver disease | 135,291 | 783 | <0.0001 | 786 | 783 | 0.93 |
| hyperthyroidism | 21,562 | 118 | <0.0001 | 99 | 118 | 0.19 |
| Musculoskeletal disease | 1,070,527 | 3,867 | <0.0001 | 3,897 | 3,867 | 0.48 |
| Heart disease | 325,223 | 1,607 | <0.0001 | 1,599 | 1,607 | 0.86 |
| Lipid disorder | 670,022 | 2,255 | 0.018 | 2,259 | 2,255 | 0.94 |
| History of hospitalization | 311,613 | 2,201 | <0.0001 | 2,215 | 2,201 | 0.78 |
| Statins | 415,977 | 1,346 | 0.94 | 1,342 | 1,346 | 0.93 |
| Aromatase inhibitors | 18,522 | 324 | <0.0001 | 311 | 324 | 0.59 |
| Levofloxacin | 509,511 | 2,705 | <0.0001 | 2,716 | 2,705 | 0.83 |
| Ciprofloxacin | 1,035,559 | 2,287 | <0.0001 | 2,280 | 2,287 | 0.89 |

| (b) |  |  |
| --- | --- | --- |
| Matched fluoroquinolone cohort with dexamethasone | Number of patients | Proportion (%) |
| C34 (Malignant neoplasm of bronchus and lung) | 225 | 0.38 |
| C25 (Malignant neoplasm of pancreas) | 60 | 0.37 |
| C90 (Multiple myeloma and malignant plasma cell neoplasms) | 78 | 0.34 |
| C15 (Malignant neoplasm of esophagus) | 21 | 0.25 |
| C79 (Secondary malignant neoplasm of other and unspecified sites) | 225 | 0.24 |
| C16 (Malignant neoplasm of stomach) | 21 | 0.21 |
| C78 (Secondary malignant neoplasm of respiratory and digestive organs) | 142 | 0.20 |
| C50 (Malignant neoplasm of breast) | 483 | 0.17 |
| Z17 (Estrogen receptor status) | 242 | 0.16 |
| C56 (Malignant neoplasm of ovary) | 38 | 0.15 |

| (c) | | | |
| --- | --- | --- | --- |
| Matched fluoroquinolone cohort | Without dexamethasone | With dexamethasone | |
|  | Fluoroquinolone | Fluoroquinolone | Dexamethasone |
| Daily dose* | 1 (1–1) | 1 (1–1) | 6.67 (4–10) |
| Cumulative dose (mg) | 6,000 (3,750–10,000) | 10,000 (5,250–20,000) | 80 (24–240) |
| Administration period (day) | 10 (7–13) | 14 (8–24) | 12 (5–30) |

***** DDD for fluoroquinolone, mg for dexamethasone

| (d) |  |  |
| --- | --- | --- |
| Matched fluoroquinolone cohort (each *n* = 5,063) | Without  dexamethasone | With  dexamethasone |
| Number of tendinopathy cases in 60 days (%) | 38 (0.75%) | 22 (0.43%) |
| M654 (Radial styloid tenosynovitis) | 2 (0.04%) | 1 (0.02%) |
| M658 (Other synovitis and tenosynovitis) | 4 (0.08%) | 0 |
| M659 (Synovitis and tenosynovitis, unspecified) | 3 (0.06%) | 1 (0.02%) |
| M662 (Spontaneous rupture of extensor tendons) | 1 (0.02%) | 0 |
| M751 (Rotator cuff tear or rupture, not specified as traumatic) | 4 (0.08%) | 0 |
| M752 (Bicipital tendinitis) | 2 (0.04%) | 1 (0.02%) |
| M754 (Impingement syndrome of shoulder) | 9 (0.18%) | 6 (0.12%) |
| M758 (Other shoulder lesions) | 2 (0.04%) | 0 |
| M760 (Gluteal tendinitis) | 0 | 1 (0.02%) |
| M765 (Patellar tendinitis) | 0 | 2 (0.04%) |
| M766 (Achilles tendinitis) | 2 (0.04%) | 1 (0.02%) |
| M767 (Peroneal tendinitis) | 1 (0.02%) | 1 (0.02%) |
| M768 (Other specified enthesopathies of lower limb, excluding foot) | 0 | 2 (0.04%) |
| M771 (Lateral epicondylitis) | 4 (0.08%) | 4 (0.08%) |
| M775 (Other enthesopathy of foot and ankle) | 2 (0.04%) | 0 |
| M778 (Other enthesopathies, not elsewhere classified) | 1 (0.02%) | 1 (0.02%) |
| M779 (Enthesopathy, unspecified) | 1 (0.02%) | 1 (0.02%) |
| S860 (Injury of Achilles tendon) | 1 (0.02%) | 0 |

**Table S6**. **Patient backgrounds, diagnoses accompanied with dexamethasone, daily and cumulative doses, and details of tendinopathy in the original and matched cohorts aged 65 or older.** (a) The number of patients for each confounding factor. (b) The number of patients and their percentage with respect to the total number of patients in each ICD-10 class. Top 10 ICD-10 classes by percentage with more than 10 patients are listed. (c) The median value and interquartile range (IQR). (d) The number of patients and their proportion with respect to the total number in the cohort.

| (a) |  |  | |  |  | | |  | | |  |  |
| --- | --- | --- | --- | --- | --- | --- | --- | --- | --- | --- | --- | --- |
| Population  aged ≥65 | Before matching | | | | After matching | | | | | | |  |
|  | Without  dexamethasone | | With  dexamethasone | *p* value | Without  dexamethasone | | With  dexamethasone | | | *p* value | |  |
| Total | 1,538,993 | | 5,232 | – | 5,232 | | 5,232 | | | – | |  |
| Median age (IQR) | 73 (68–80) | | 73 (68–78) | <0.0001 | 73 (68–78) | | 73 (68–78) | | | 0.74 | |  |
| Female | 850,048 | | 2,947 | 0.11 | 2,943 | | 2,947 | | | 0.94 | |  |
| Renal Failure | 241,732 | | 1,062 | <0.0001 | 1,021 | | 1,062 | | | 0.32 | |  |
| Obesity | 246,523 | | 923 | 0.0014 | 902 | | 923 | | | 0.59 | |  |
| Diabetes | 429,765 | | 1,541 | 0.014 | 1,515 | | 1,541 | | | 0.58 | |  |
| Rheumatoid arthritis | 40,105 | | 181 | 0.0001 | 165 | | 181 | | | 0.38 | |  |
| Gout | 60,197 | | 201 | 0.67 | 175 | | 201 | | | 0.17 | |  |
| Cancer | 363,457 | | 3,219 | <0.0001 | 3,215 | | 3,219 | | | 0.94 | |  |
| Liver disease | 83,810 | | 739 | <0.0001 | 698 | | 739 | | | 0.24 | |  |
| hyperthyroidism | 18,323 | | 87 | 0.0017 | 69 | | 87 | | | 0.15 | |  |
| Musculoskeletal disease | 1,088,700 | | 4,321 | <0.0001 | 4,328 | | 4,321 | | | 0.86 | |  |
| Heart disease | 687,192 | | 3,009 | <0.0001 | 3,025 | | 3,009 | | | 0.75 | |  |
| Lipid disorder | 998,658 | | 3,503 | 0.0018 | 3,526 | | 3,503 | | | 0.63 | |  |
| Hospitalization | 359,758 | | 2,848 | <0.0001 | 2,850 | | 2,848 | | | 0.97 | |  |
| Statins | 795,599 | | 2,927 | <0.0001 | 2,937 | | 2,927 | | | 0.84 | |  |
| Aromatase inhibitors | 20,164 | | 218 | <0.0001 | 218 | | 218 | | | 1.00 | |  |
| Fluoroquinolones | 291,181 | | 1,865 | <0.0001 | 1,862 | | 1,865 | | | 0.95 | |  |
| (b) | | | | | |  | | |  | | | |
| Matched older adult cohort with dexamethasone | | | | | | Number of patients | | | Proportion (%) | | | |
| C45 (Mesothelioma) | | | | | | 17 | | | 1.44 | | | |
| C90 (Multiple myeloma and malignant plasma cell neoplasms) | | | | | | 196 | | | 0.86 | | | |
| C34 (Malignant neoplasm of bronchus and lung) | | | | | | 366 | | | 0.62 | | | |
| C79 (Secondary malignant neoplasm of other and unspecified sites) | | | | | | 330 | | | 0.36 | | | |
| C25 (Malignant neoplasm of pancreas) | | | | | | 57 | | | 0.35 | | | |
| C57 (Malignant neoplasm of other and unspecified female genital organs) | | | | | | 11 | | | 0.25 | | | |
| C16 (Malignant neoplasm of stomach) | | | | | | 24 | | | 0.24 | | | |
| C56 (Malignant neoplasm of ovary) | | | | | | 58 | | | 0.24 | | | |
| C15 (Malignant neoplasm of esophagus) | | | | | | 19 | | | 0.23 | | | |
| C78 (Secondary malignant neoplasm of respiratory and digestive organs) | | | | | | 126 | | | 0.18 | | | |

| (c) |  |
| --- | --- |
| Matched older adult cohort with dexamethasone | Dexamethasone |
|  |  |
| Daily dose (mg) | 6  (3–10) |
| Cumulative dose (mg) | 72  (20–200) |
| Administration period (day) | 10  (4–30) |

| (d) |  |  |
| --- | --- | --- |
| Matched older adult cohort (each *n* = 5,232) | Without  dexamethasone | With  dexamethasone |
| Number of tendinopathy cases in 180 days (%) | 112 (2.14%) | 76 (1.45%) |
| M654 (Radial styloid tenosynovitis) | 5 (0.10%) | 2 (0.04%) |
| M658 (Other synovitis and tenosynovitis) | 10 (0.19%) | 5 (0.10%) |
| M659 (Synovitis and tenosynovitis, unspecified) | 2 (0.04%) | 1 (0.02%) |
| M662 (Spontaneous rupture of extensor tendons) | 1 (0.02%) | 0 |
| M663 (Spontaneous rupture of flexor tendons) | 1 (0.02%) | 0 |
| M668 (Spontaneous rupture of other tendons) | 0 | 1 (0.02%) |
| M751 (Rotator cuff tear or rupture, not specified as traumatic) | 18 (0.34%) | 15 (0.29%) |
| M752 (Bicipital tendinitis) | 8 (0.15%) | 1 (0.02%) |
| M754 (Impingement syndrome of shoulder) | 14 (0.27%) | 12 (0.23%) |
| M758 (Other shoulder lesions) | 9 (0.17%) | 7 (0.13%) |
| M759 (Shoulder lesion, unspecified) | 1 (0.02%) | 0 |
| M760 (Gluteal tendinitis) | 3 (0.06%) | 3 (0.06%) |
| M765 (Patellar tendinitis) | 2 (0.04%) | 1 (0.02%) |
| M766 (Achilles tendinitis) | 5 (0.10%) | 1 (0.02%) |
| M767 (Peroneal tendinitis) | 3 (0.06%) | 4 (0.08%) |
| M768 (Other specified enthesopathies of lower limb, excluding foot) | 10 (0.19%) | 5 (0.10%) |
| M769 (Unspecified enthesopathy, lower limb, excluding foot) | 1 (0.02%) | 0 |
| M771 (Lateral epicondylitis) | 3 (0.06%) | 3 (0.06%) |
| M775 (Other enthesopathy of foot and ankle) | 5 (0.10%) | 9 (0.17%) |
| M778 (Other enthesopathies, not elsewhere classified) | 3 (0.06%) | 0 |
| M779 (Enthesopathy, unspecified) | 8 (0.15%) | 2 (0.04%) |
| S860 (Injury of Achilles tendon) | 0 | 4 (0.08%) |
